# Integrated glycoproteomics reveals site-specific N-glycosylation defects in phosphomannomutase two congenital disorder of glycosylation

**DOI:** 10.1016/j.jbc.2026.113469

**Published:** 2026-08-19

**Authors:** Jonas Nilsson, Ekaterina Mirgorodskaya, Carina Sihlbom Wallem, Fredrik Noborn, Erik A. Eklund, Göran Larson, Maria Blomqvist

**Affiliations:** 1 Proteomics Core Facility (PCF) and BioMS, Sahlgrenska Academy, University of Gothenburg, Gothenburg, Sweden; 2 Glycoproteomics and MS Proteomics, Science for Life Laboratory (SciLifeLab), University of Gothenburg, Gothenburg, Sweden; 3 Department of Laboratory Medicine, Institute of Biomedicine, University of Gothenburg, Gothenburg, Sweden; 4 Section for Pediatrics, Department of Clinical Sciences, Lund University, Lund, Sweden; 5 VO Barnmedicin, Skåne University Hospital, Lund, Sweden; 6 Department of Clinical Chemistry, Sahlgrenska University Hospital, Gothenburg, Region Västra Götaland, Sweden

**Keywords:** biomarker, carbohydrate metabolism, congenital disorders of glycosylation (CDG), glycomics, glycopeptide, glycoproteomics, inborn error of metabolism, mass spectrometry (MS), N-linked glycosylation, PMM2-CDG, spectral counting

## Abstract

The routine diagnostic pathway for congenital disorders of glycosylation (CDG) relies on the analysis of carbohydrate-deficient transferrin in serum, a test that incompletely captures defects in N-linked glycosylation and may also yield false-negative results even in classical N-glycosylation disorders such as PMM2-CDG. To explore molecular features of PMM2-CDG pathogenesis and identify potential biomarker candidates, we applied an integrated semiquantitative LC–MS/MS glycoproteomic workflow to a well-characterized cohort of individuals with PMM2-CDG (n = 7), alongside controls (n = 9). The workflow combines the analysis of *N*-glycopeptides and corresponding non-glycosylated peptides in serum and plasma. Distinct disease-associated glycosylation patterns were observed in PMM2-CDG patients. Elevated levels of immature (paucimannose-like) *N*-glycans were detected at specific sites, including GlcNAc(2)Man(4) at Asn-85 of complement component 3, GlcNAc(2)Man(3) at Asn-869 of α-2-macroglobulin, and GlcNAc(2)Man(5) at Asn-226 of complement component 4. In parallel, approximately 50 peptides with unoccupied *N*-glycosylation sequons, including peptides from transferrin, were recurrently identified in CDG patient samples, indicating widespread reductions in *N*-glycosylation site occupancy. Together, this small cohort-based glycoproteomic analysis reveals coherent, site-specific disruptions in protein glycosylation that reflect underlying defects in mannose metabolism in PMM2-CDG. These molecular features define candidate glycophenotypes with potential utility as biomarkers for CDG subtype discrimination, disease stratification, and assessment of disease severity in future studies.

Congenital disorders of glycosylation (CDGs) are a group of inherited metabolic disorders caused by inadequate synthesis, processing or transport of glycans attached to proteins and lipids. The disorders vary in disease severity and present a wide clinical spectrum, often presenting a multisystem pathology with neurological involvement (1). Advances in next-generation sequencing over the past 10 to 15 years have led to a rapid expansion in the number of identified CDGs. Currently, approximately 190 genes are known to cause a CDG, accounting for more than 200 distinct clinical presentations (2). Each disorder is designated using the format *gene name-CDG* (3). The CDGs can be classified as follows: (i) Disorders of *N*-linked protein glycosylation, (ii) Disorders of *O*-linked protein glycosylation, (iii) Disorders of lipid glycosylation, (iv) Disorders of monosaccharide synthesis and interconversion, (v) Disorders of nucleotide sugar synthesis and transport, (vi) Disorders of vesicular trafficking, (vii) Disorders of multiple glycosylation pathways, and (viii) Disorders of glycoprotein/glycan degradation (2).

The most common form of CDG is by far phosphomannomutase two deficiency (PMM2-CDG), with an estimated incidence of one in 33,576 in North America and Europe combined, corresponding to approximately 300 affected live births per year in these continents (4). PMM2-CDG is an autosomal recessive disorder caused by biallelic pathogenic variants in the PMM2 gene, with more than a hundred disease-causing variants reported worldwide. However, several recurrent variants exhibit population-specific enrichment, such as the p.Arg141His (R141H) variant, which is particularly frequent among individuals of European ancestry and constitutes the most frequently identified pathogenic allele in PMM2-CDG cohorts (4). The phenotypic spectrum ranges from early lethality to survival into adulthood, sometimes with normal or near-normal cognition (5). Major clinical features include neonatal hypoglycemia, liver disease, muscular hypotonia, developmental delay and intellectual disability, epilepsy, stroke-like episodes, retinitis pigmentosa, and polyneuropathy (1, 6). The enzymatic step adjacent to PMM2, catalyzed by phosphomannose isomerase (MPI), is affected in MPI-CDG. MPI-CDG is considerably rarer than PMM2-CDG and is likewise inherited in an autosomal recessive manner. In this form of CDG, neurological involvement is absent, and the major features include hypoglycemia, protein-losing enteropathy, and liver disease. Notably, MPI-CDG is one of the few treatable CDGs; oral mannose supplementation alleviates most symptoms (7).

When a CDG is suspected, the current first-line diagnostic test is serum transferrin (TF) glycoform analysis, performed using High-Performance Liquid Chromatography (HPLC), isoelectric focusing, capillary electrophoresis or mass spectrometry of the intact glycoprotein (8). In the previous CDG nomenclature (9), protein glycosylation defects were classified into two types depending on the biochemical pathway that was affected. Defects in glycan assembly occurring in the endoplasmic reticulum (ER) produced a type I TF pattern and were designated CDG type I. In contrast, defects affecting ER-Golgi transport or subsequent glycan processing in the Golgi apparatus resulted in a type II TF pattern and were classified as CDG type II. However, such TF analysis only covers the *N*-linked glycosylation defects (approximately half of all the CDGs), and there is thus a clinical need for novel diagnostic tests that can identify a wider range of CDG subtypes. Although whole-exome and -genome sequencing have greatly improved diagnostic yield, they also identify numerous novel genetic variants, including variants of uncertain significance (VUS), which complicates the clinical interpretation. Consequently, additional glycan-based biomarkers are required to enable definitive diagnosis within this disease group. Furthermore, as new therapies are emerging, specific biomarkers for each CDG are also urgently needed for clinical study stratification and treatment follow-up.

Recently, glycoproteomic approaches for identifying site-specific glycosylation in complex biological samples such as plasma have advanced significantly (10, 11, 12, 13, 14, 15, 16), emerging as a powerful approach for clinical diagnostics. Glycomics as well as glycoproteomics have been applied in biomarker studies across a broad range of diseases, including Alzheimer’s disease and other neuroinflammatory conditions, myocardial infarction, diabetes and venous thromboembolism risk stratification (17, 18, 19, 20, 21, 22). For rare diseases such as CDG, glycomics (23, 24, 25) and glycoproteomic analytical approaches of site-specific glycosylation changes (26, 27, 28) have emerged as strategies that may improve the specificity of various CDG biomarkers. Interestingly, other plasma glycoproteins, such as immunoglobulins and complement 4-A and 4-B (C4), have been suggested as possible biomarkers alongside TF analysis (28, 29, 30).

In this study, we aimed to apply a discovery-driven glycoproteomic workflow to advance the structural characterizations of PMM2-CDG plasma proteins, to identify potential biomarker candidates and to provide new insights into disease mechanisms. We primarily employed spectral counting-based relative quantification (31, 32, 33), an approach well suited for exploratory glycoproteomic analyses, as it enables the identification of consistent differences in glycopeptide detection and glycoform distributions within and across samples. For selected features, precursor ion intensity measurements were additionally used to support the spectral counting–based findings and to provide a foundation for the development of future targeted quantification strategies.

## Results

### Cohort characteristics

Seven individuals with PMM2-CDG and two individuals with MPI-CDG were included in this study. The salient features of the patients are summarized in Table 1. Serum TF glycoform analysis by HPLC and/or LC-MS/MS for detecting aberrant glycosylation pattern was performed as part of the standard diagnostic procedure for CDG alongside with genetic analysis. Serum TF normally contains two biantennary sialylated *N*-glycans at Asn-432 and Asn-630, resulting in four sialic acids per molecule (tetrasialo-transferrin). TF analysis at the time of diagnosis showed a type I CDG-profile (increased proportion of di- and a-sialo-transferrin, where one or two of the entire *N*-glycans were lacking), in six out of the 7 PMM2-CDG patients, as well as in the two MPI-CDG patients. Furthermore, two PMM2-CDG patients were analyzed for PMM2 activity in leucocytes (34) to confirm diagnosis (Table 1). Along with the above patient samples, plasma from nine control individuals (3 months to 58 years of age, Table S1) were included for the in-depth analysis of their plasma or serum glycoproteome.

**Table 1:** Salient features of affected individuals

Gene defect (patient number)	Sex	Genotype	Transferrin profile (HPLC)	Sample matrix	Age at sampling	PMM2 activity. Leucocytes (reference interval 39.5–83.9 μkatal/kg protein)
PMM2-CDG_1	M	PMM2:c.357C>A, p.(Phe119Leu) PMM2:c.422G>A, p.(Arg141His)	Type I^a^	plasma	10 months	25 μkatal/kg protein^b^
PMM2-CDG_2	M	PMM2:c.357C>A, p.(Phe119Leu) PMM2:c.422G>A, p.(Arg141His)	N/A	plasma	4.5 months	Below detection limit (<3.7 μkatal/kg protein)
PMM2-CDG_3	M	PMM2:c.357C>A, p.(Phe119Leu) HOM (NM_000303.3)	Type I^a^	plasma	7 months	N/A
PMM2-CDG_4	F	PMM2:c.357C>A, p.(Phe119Leu) PMM2:c.422G>A, p.(Arg141His) (NM_000303.3)	Type I^a^	plasma	39 years	N/A
PMM2-CDG_5	M	PMM2:c.357C>A, p.(Phe119Leu) PMM2:c.422G>A, p.(Arg141His) (NM_000303.3)	Type I^a^	plasma	45 years	N/A
PMM2-CDG_6	M	PMM2:c.422G>A, p.(Arg141His) PMM2:c.554A>G, p. (Asp185Gly) (NM_000303.3)	Type I^a^	plasma	6 years 4 months	N/A
PMM2-CDG_7	F	PMM2:c.32_33delinsGT, p.(Phe11Cys) PMM2:c.422G>A, p.(Arg141His) (NM_000303.2)	Type I^a^	serum	16 years	N/A
MPI-CDG_1	F	N/A	Type I^a^	serum	N/A	N/A
MPI-CDG_2	M	MPI:c.656G>A p.(Arg219Gln) MPI:c.1252C>T p.(Arg418Cys) (NM_002435.2)	Type I^a^	serum	3 years 11 months	N/A

a asialo- and disialo-transferrin.

b Non fasting, HOM; homozygous.

### Glycoproteomic methodology

**Figure 1:**
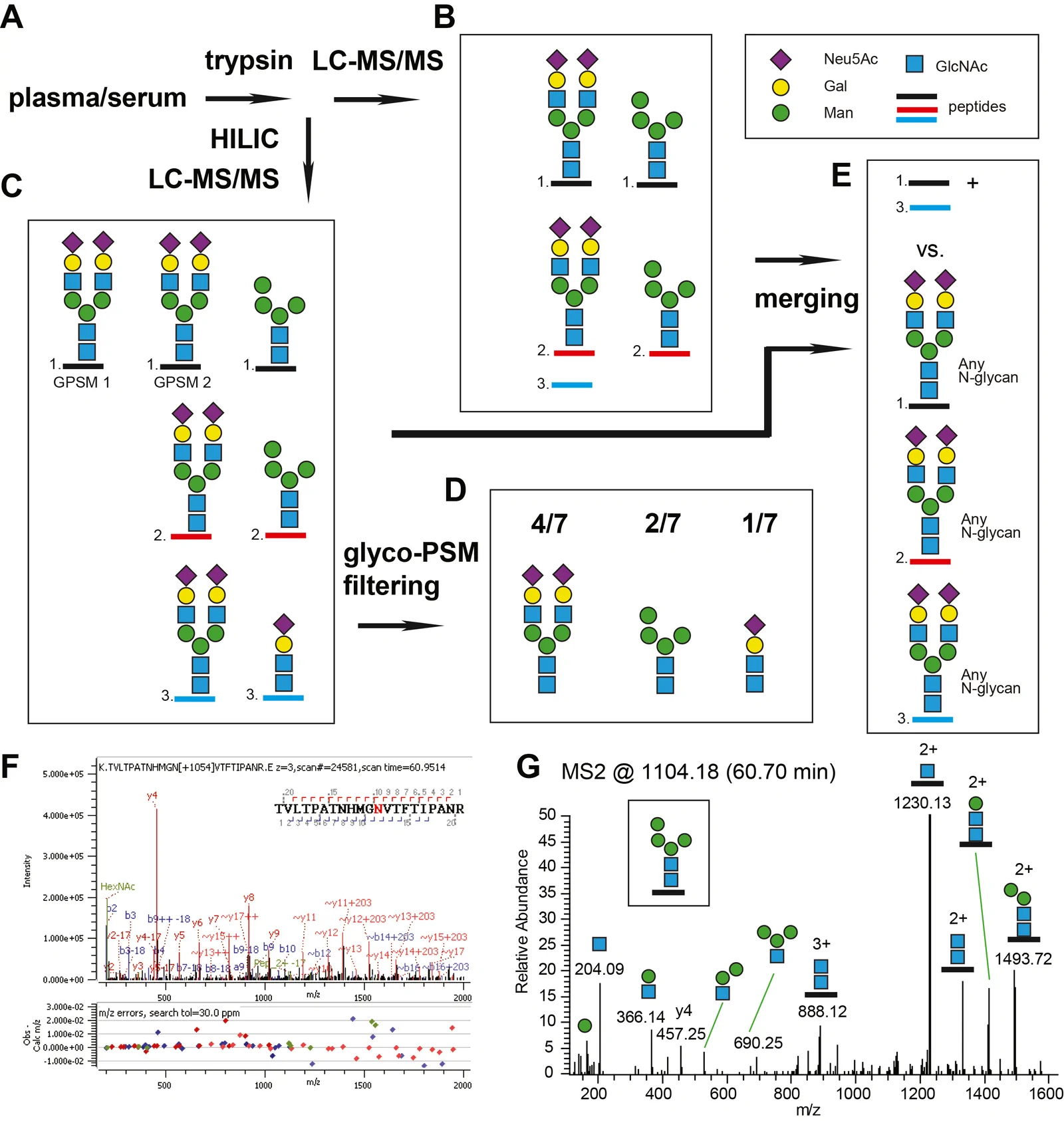
**The glycoproteomic workflow for the identification and relative quantification of glycopeptides.***A*, plasma/serum samples were digested with trypsin, whereafter one aliquot (*B*) was directly used for LC-MS/MS, and one aliquot (*C*) underwent HILIC purification of glycopeptides before LC-MS/MS. *D*, scripts were used to compile identical GPSMs and their percentages relative of all glycoforms (here represented by ratios from the GPSMs in *panel C*). *E*, the non-HILIC and HILIC datasets were merged to identify non-glycosylated peptides (*straight lines*) with corresponding peptide sequences also identified in glycosylated form(s). *F*, the glycoproteomic data analysis was performed using Byonic software. *G*, verification of glycan structures were performed manually at lower fragmentation energies. The example *N-*glycopeptide in *F* and *G* is from residues 74 to 94 of complement 3 (C3), containing the Asn-85 glycosylation site, carrying the GlcNAc(2)Man(4) (M4) structure.

Following trypsin digestion of the samples (Fig. 1*A*), each sample was split into two aliquots. One aliquot was used directly for LC-MS/MS (Fig. 1*B*) while the other underwent enrichment of glycopeptides using zwitterionic hydrophilic interaction liquid chromatography (ZIC-HILIC) prior to LC-MS/MS analysis (Fig. 1*C*). To assay the PMM2-CDG and control samples, the number of glycopeptide spectrum matches (GPSMs) for specific glycopeptides and glycan structures was used for the relative quantification of glycoforms within each sample and then compared across samples (Fig. 1*D*). Also, a combined analysis of the non-HILIC (Fig. 1*B*) and the HILIC enriched (Fig. 1*C*) datasets was performed to identify pairs of non-glycosylated peptides and their corresponding *N*-glycopeptides (Fig. 1*E*). For LC-MS/MS, higher-energy collision induced dissociation (HCD) was performed using a relatively high normalized collision energy (NCE) of 38% to maximize the presence of b- and y-ions, including deglycosylated b-/y-ions, which are essential for the high-quality spectrum matching using glycoproteomic search engines (Figs. 1*F* and S1). Furthermore, while the LC elution times of glycopeptide analytes typically exceeded 20 s, a relatively short exclusion time of 12 s was applied during the data-dependent acquisition. This enabled repeated fragmentation events of precursor ions within their elution time window. Since more abundant analytes exceeded the intensity threshold for triggering MS/MS for a prolonged time, often up to 1 min, the peptide spectrum matches (PSM)-counts for each analyte reflects their relative concentrations in the sample (Fig. S1). Finally, lower NCEs of 20% and 30% were used in separate LC-MS/MS experiments to manually verify the glycan structures of glycopeptides of particular interest (Figs. 1*G* and S1).

### Glycoproteomic data

**Figure 2:**
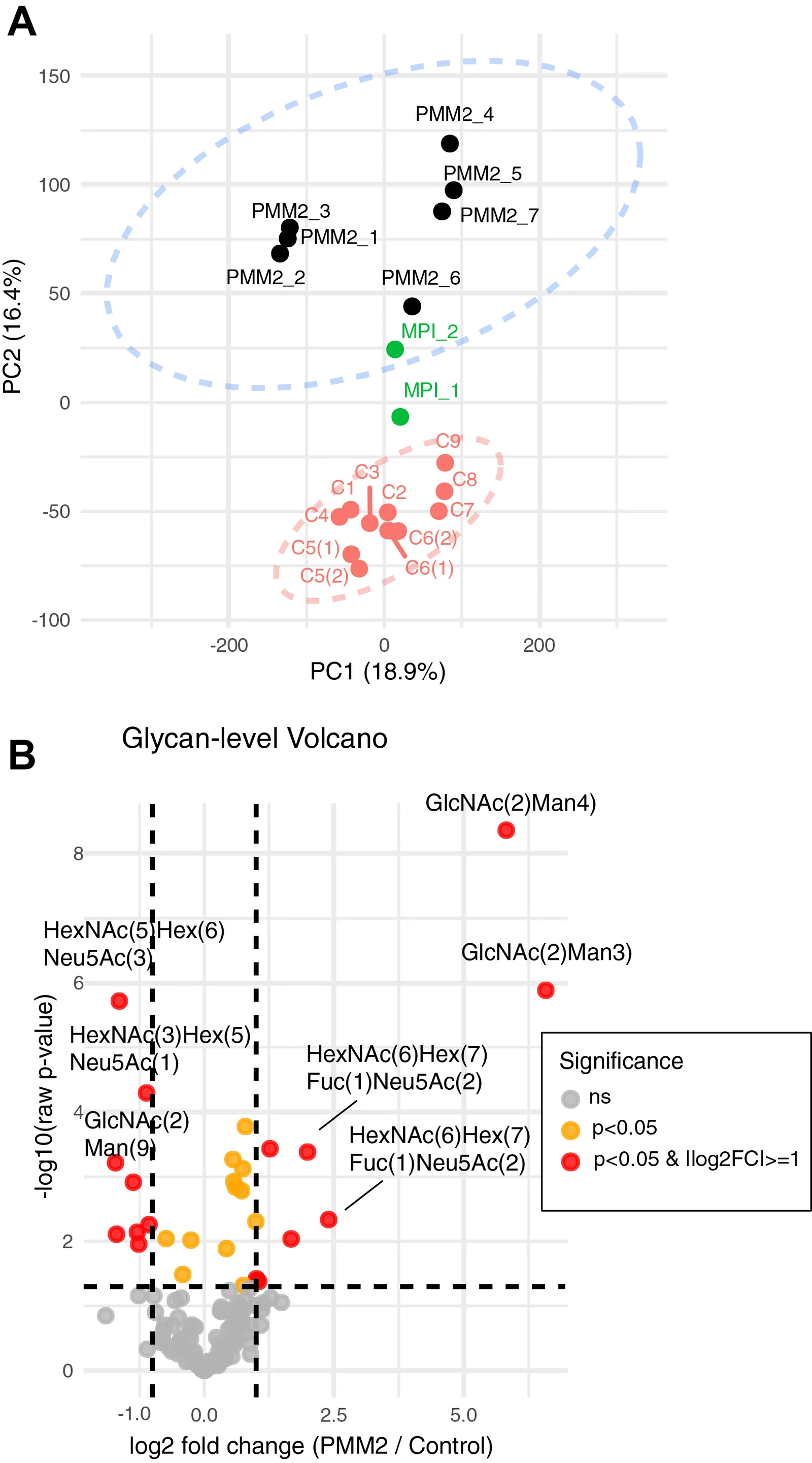
**PCA and volcano plot analysis of the glycoproteomic data.***A*, the PCA plot of the HILIC-enriched glycopeptide datasets. The top 300 glycopeptides with the strongest contributions to the PC2 dimension are listed in Table S2. *B*, the volcano plot of the identified glycans, irrespective of their protein glycosylation sites. Two-sided Welch’s t-tests were implemented. Control samples five and six were prepared and analyzed by LC-MS/MS on two different occasions.

Using our quantitative glycoproteomic methodology, an average of 981 (18% CV) and 921 (21% CV) unique *N*-glycopeptides were identified from the patient and control HILIC-enriched samples (n = 16), respectively, and together 2588 unique glycopeptides were identified. These glycopeptides covered 286 glycosylation sites from 142 different proteins. Thus, multiple glycan structures were identified for most glycosylation sites, and many of the glycoproteins contained several glycosylation sites (Fig. S2). To assay the global changes in protein glycosylation and to identify biomarker candidates for further evaluation, we first applied a principal component analysis (PCA) to the HILIC MS/MS datasets, that revealed clear stratification of PMM2 and control samples in line with systematic shifts in the glycopeptide profiles (Fig. 2*A*, Table S2). The PCA positioned the two MPI-CDG patients just in between the two major groups and demonstrated that the control group clustered well together suggesting that their individual glycan profiles were relatively similar. The results showed comparable performance between the analytical runs, with very little variation observed for the control samples in the PCA analysis; that is, C5(1) clustered closely with C5(2), and similarly C6(1) clustered closely with C6(2) (Fig. 2*A*). Furthermore, a volcano plot of the glycan profiles, irrespective of their glycopeptide origins, provided a glycomic view of the glycan alterations, and identified the GlcNAc(2)Man(3) glycan, from here on called Man-3 (M3), and GlcNAc(2)Man(4) [Man-4 (M4)] to be significantly increased in the PMM2-CDG samples (Fig. 2*B*). We refer to M3 and M4 as paucimannose-like glycans, since they contain fewer Man residues than the typical M5–M10 oligomannose glycans observed in human plasma (21). This terminology is used descriptively; the detected M3 and M4 compositions do not necessarily represent classical paucimannose glycans, which often denote extensively trimmed M1–M3 structures, frequently with core fucosylation (35, 36). Instead, in PMM2-CDG, these compositions may alternatively arise from transfer of immature lipid-linked oligosaccharide (LLO)-derived glycans (Fig. S3).

### Oligomannose N-glycan microheterogeneity of plasma glycoproteins in PMM2-and MPI-CDG

**Figure 3:**
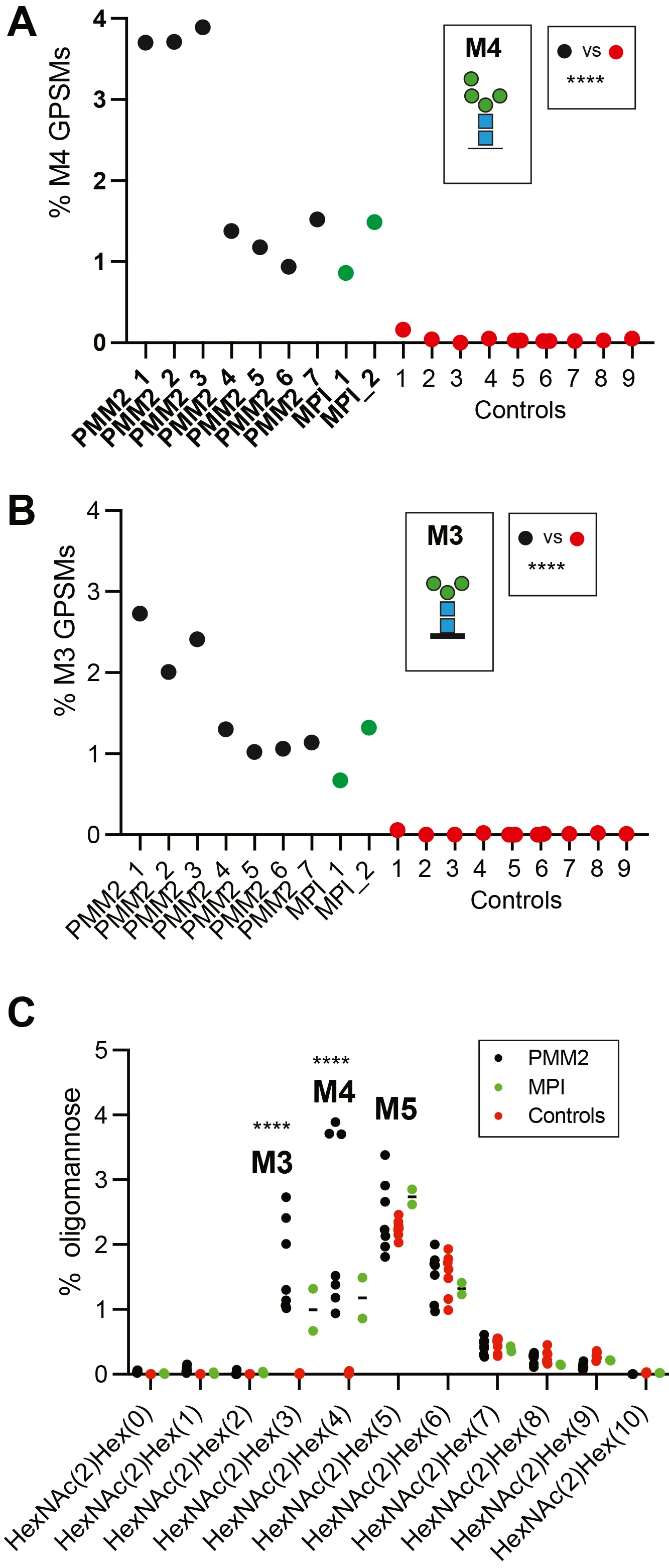
**Glycopeptide spectrum matches (GPSMs) of oligomannose glycoforms.***A*, the percentage of GPSMs for the oligomannose-4 (M4) glycoform vs. all other *N*- glycoforms. *B*, the percentage of GPSMs for the oligomannose-3 (M3) glycoform vs. all other *N*-glycoforms. *C*, the mean relative percentages of the GlcNAc(2)Man(0) through GlcNAc(2)Man(10) glycoforms. PMM2-CDG (n = 7, *black bars*), MPI-CDG (n = 2, *green bars*), and control individuals (n = 9, *red bars*). Mean values (*vertical line*) and *p* < 0.0001 values (∗∗∗∗) of PMM2 vs controls are indicated. A two-sided unpaired *t* test was implemented for the PMM2-CDG and control samples 2 to 8 (n = 7).

Next, we focused on the oligomannose glycosylation of the individual patient and control samples and found a pronounced, but varying, enrichment of the M3 and M4 *N-*glycans in the disease groups compared to controls (Fig. 3, *A* and *B*). Significant relative increases of the M3 and M4 *N-*glycopeptide mean values were present in the group of PMM2-CDG patients (*p* < 0.0001). The group of MPI-CDGs showed the same trend but due to the small number of patients, statistical calculations were not performed. The proportion of GPSMs corresponding to M4 relative to all other glycoforms exceeded 3% in the three PMM2-CDG patients younger than 1 year, whereas the four older patients (6–45 years) showed levels of 0.8 to 1.5%. These values were similar to those observed in the two MPI-CDG patients, but clearly distinct from controls, all of whom had M4 glycopeptide levels below 0.1%. In contrast, analysis of the GlcNAc(2)Man(5) (M5) to GlcNAc(2)Man(10) (M10) glycoforms (Fig. 3*C*) showed a relative increase of the larger oligomannose glycoforms in the controls, for which the M5 and M6 glycans dominated.

### Site-specific alterations in oligomannose N-glycosylation of selected glycoproteins

**Figure 4:**
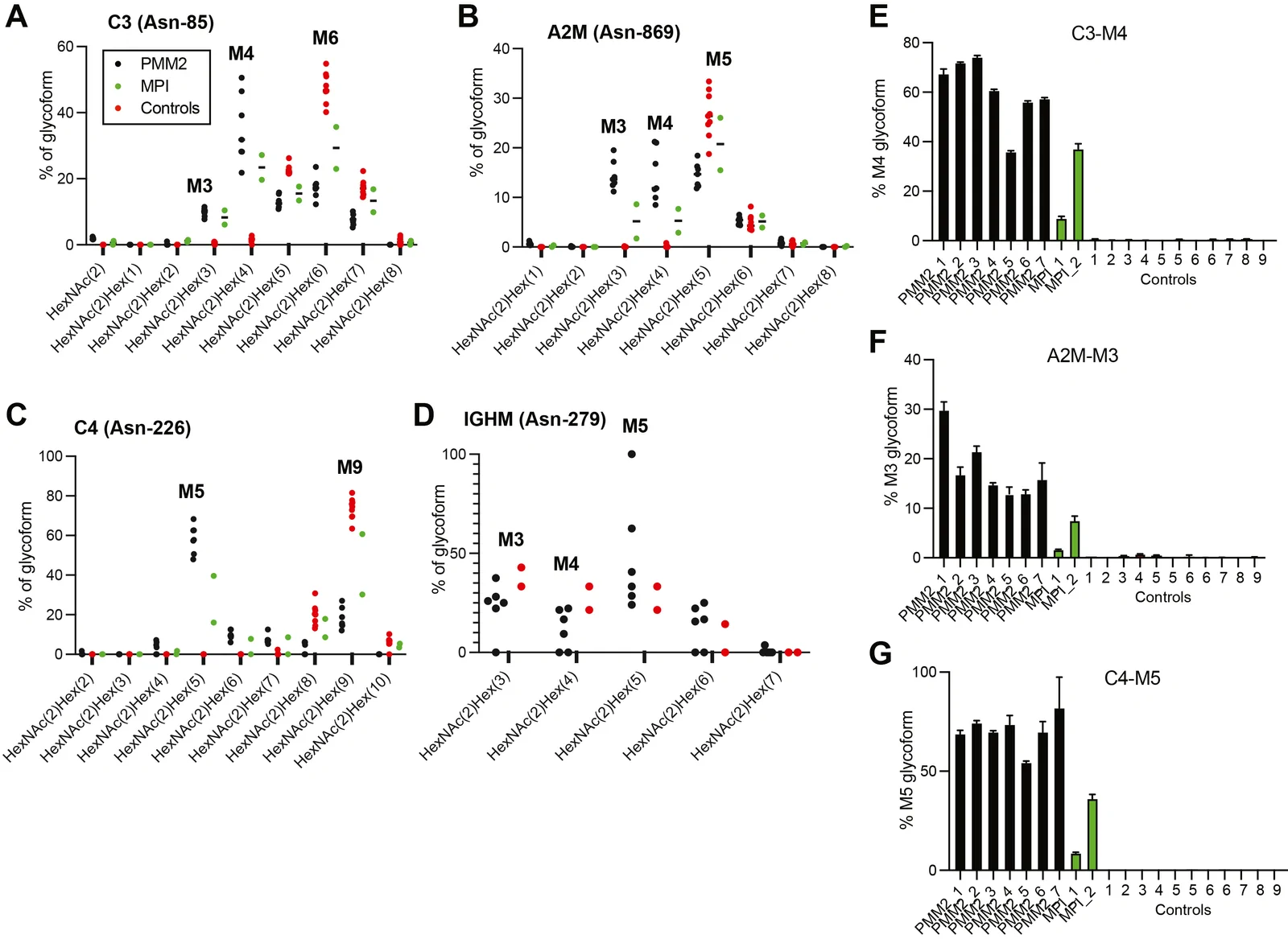
**The relative abundances of oligomannose glycoforms of selected peptides.** The graphs points illustrate GPSM results from single samples for (*A*) Asn-85 of complement 3 (C3); (*B*) Asn-869 of alpha-2-macroglobulin (A2M); (*C*) Asn-226 of complement 4-A and 4-B (C4); and (*D*) Asn-279 of immunoglobulin heavy constant mu (IGHM). The IGHM sequence THTNISESHPNATFSAVGEASICEDDWNSGER was only found glycosylated at the second *N*-consensus site in the sequence (both sites *underlined*) in the CDG patients. The relative abundances are illustrated for (*E*) the C3-M4 glycoform, (*F*) the A2M-M3 glycoform and for (*G*) the C4-M5 glycoform, as measured using precursor ion intensities of those glycoforms *versus* all other glycoforms of the corresponding glycopeptide. The error bars show the standard deviation for triple injections for the CDG samples and double injections for the control samples. Control samples five and six were prepared and analyzed at two separate occasions.

Given the pronounced alterations in overall microheterogeneity of the oligomannose glycoforms observed in the patient cohort, we additionally investigated these glycan discrepancies at the level of individual glycopeptides. Thus, data analysis was focused on glycopeptides that contain oligomannose structures. For one of these glycoproteins, complement 3 (C3), the relative percentage of oligomannose glycopeptides containing Asn-85 showed a significant increase (*p* < 0.0001), with the M4 glycoform predominating compared with controls, in which M6 was dominant (Fig. 4*A*). These findings suggest that the C3-M4 glycopeptide may serve as a biomarker for PMM2-CDG and possibly for MPI-CDG. A relative increase in the M4 glycoform was also observed at Asn-869 of alpha-2-macroglobulin (A2M), along with a pronounced increase in the M3 glycoform (Fig. 4*B*). In contrast, the glycan at Asn-226 of C4 (Fig. 4*C*), was dominated by the M5 glycoform in the patient cohorts and by the M9 glycoform in the controls. Further, the M3 and M4 glycoforms were also identified in a peptide [amino acids 269–300] of immunoglobulin heavy constant mu (IGHM), which include two Asn-X-Ser/Thr (NXS/T, X ≠ Pro) consensus *N*-glycosylation motifs at Asn-279 and Asn-286 (Fig. 4*D*), where Asn-279 was identified as the glycosylation site (Fig. S4). Interestingly, no such IGHM glycopeptides were identified in the control samples. Thus, this peptide stretch may include *N*-glycans at both *N-*glycosylation sites only in healthy controls, suggesting a reduced NXS/T motif site-occupancy in the PMM2-and MPI-CDG samples. Further, in line with the identification of the M3, M4 and M5 biomarker candidates of the major oligomannose glycopeptides in plasma, we also detected less abundant glycopeptides from complement 2 (C2), complement 7 (C7), attractin and Asn-45 of IGHM (Fig. S5). For these glycopeptides, the paucimannose-like and oligomannose glycan patterns showed a trend toward increased levels of M3 and M4 glycans in patients compared with controls. In summary, the paucimannose-like and oligomannose glycosylation patterns may be useful as future disease biomarkers and were consistently identified at only 8 *N*-glycosylation sites from seven plasma proteins.

Because the M5 composition may arise both as an immature LLO-derived glycan and as a normally trimmed oligomannose structure, detection of M5 alone is not disease-specific (Fig. S3), *that is,* which mannose arm is extended or retained. We therefore inspected representative MS2 spectra of C4-M5 from PMM2-CDG samples, together with C3-M5 and A2M-M5 spectra available from both CDG and control samples, to determine whether the glycosidic fragmentation patterns could provide clues for the presence of immature *versus* trimmed oligomannose structures. No clear, reproducible differences were observed, and further structural characterization is beyond the scope of this study.

To verify the spectral count results, the relative quantifications of the most prominent oligomannose glycopeptides were measured using the signal intensities of the precursor ions. The relative abundances of C3-M4 (Fig. 4*E*), A2M-M3 (Fig. 4*F*), and C4-M5 (Fig. 4*G*), compared to those of all the identified glycopeptide precursor ions in each sample, were clearly predominant (*p* < 0.0001) in PMM2-CDG samples and, to a lesser extent, in the two MPI-CDG samples, while being scarcely detectable in controls. Again, this difference supports the use of the site-specific paucimannose-like structures as biomarker candidates for the PMM2-and possibly the PMI-CDG patients.

### Site-specific identification of an aberrant N-tetrasaccharide in PMM2-and MPI-CDG

**Figure 5:**
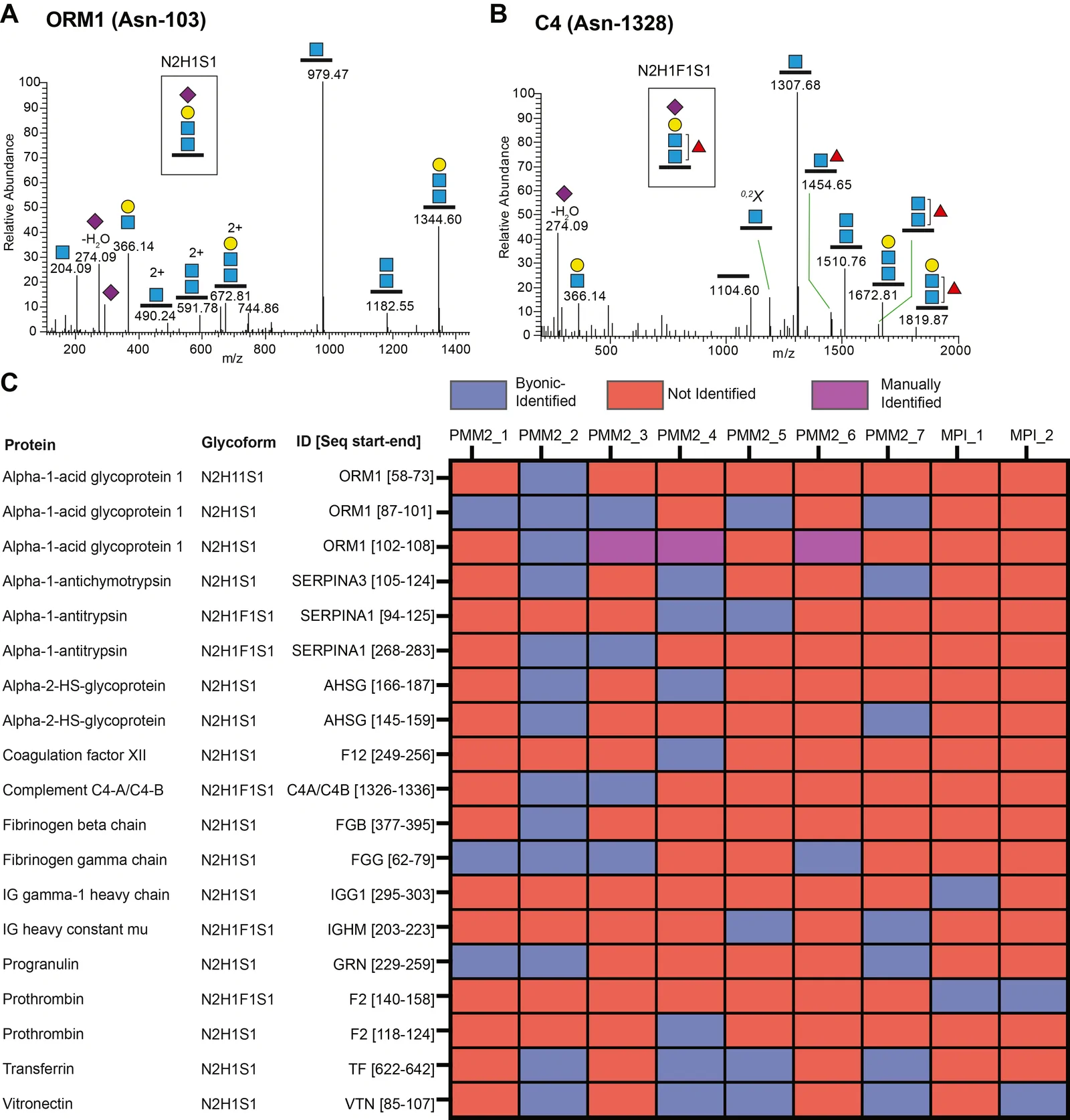
**Identification of aberrant *N*-tetra/pentasaccharides in PMM2-and MPI-CDGs.***A*, MS2 spectrum of a glycopeptide [amino acids 102–108] of alpha-1-acid glycoprotein 1 (ORM1) carrying a HexNAc(2)Hex(1)Neu5Ac(1) (N2H1S1) glycan at Asn-103. *B*, MS2 spectrum of a glycopeptide [amino acids1326–1336] of complement C4-A and C4-B (C4) carrying HexNAc(2)Hex(1)Fuc(1)Neu5Ac(1) (N2H1F1S1) at Asn-1328. *C*, summary of identified *N*-tetra/pentasaccharide glycopeptides (*i.e.* with or without a fucose) from the PMM2-and MPI-CDG patients. No such *N*-tetrasaccharide glycopeptides were identified in the control samples.

In glycomic analysis of released *N*-glycans from PMM2-and MPI-CDG samples, a novel tetrasaccharide *N*-glycan with the composition HexNAc(2)Hex(1)Neu5Ac(1) (N2H1S1) has been reported (37). We included this saccharide modification with and without fucose in our Byonic analysis. Doing so, we identified this glycan at Asn-103 of alpha-1-acid glycoprotein 1 (also known as orosomucoid, ORM1, Fig. 5*A*) and at Asn-1328 of C4 where it was additionally carrying a fucose residue (N2H1F1S1, Fig. 5*B*). The characteristic loss of 120.04 Da from the peptide + HexNAc ion, due to an ^0.2^*X*-cleavage of the *N*-glycosidically bound HexNAc, demonstrated that we were indeed observing *N-*glycosylation - as opposed to *O*-glycosylation of Ser/Thr in the peptide. The available fragment ions were consistent with a N2H1 F1S1 composition but did not allow unambiguous localization of the fucose residue. In particular, no diagnostic B-ions at *m/z* 350.15 (HexNAcFuc), *m/z* 512.20 (HexNAcHexFuc), or *m/z* 803.29 (HexNAcHexFucNeu5Ac) were observed to support fucosylation on the terminal GalGlcNAc residues. Conversely, assignment of the fucose to the core GlcNAc is complicated by the possibility of fucose migration during positive-mode collisional dissociation of protonated glycopeptide ions (38). We therefore annotate the glycan as HexNAc(2)Hex(1)Fuc(1)Neu5Ac(1), with the fucose position unresolved, and depict it with bracketed fucose position. The low abundance of these glycopeptides precluded further structural characterization. The identified *N-*tetrasaccharide glycopeptides were found in three sites from ORM1, two sites from alpha-1-antitrypsin and alpha-2-HS-glycoprotein and at Asn-630 from TF (Fig. 5*C*). The largest number of identifications was 13, made in PMM2-CDG patient 2, followed by eight in PMM2-CDG patient four and seven in PMM2-CDG patient 7. The MPI-CDG samples provided only two identifications each. No identification of the *N*-tetrasaccharides was made in any of the control samples, indicating its aberrant presence in the CDG samples.

### Increased presence of peptides with unoccupied N-glycosylation sites in PMM2-and MPI-CDG

The experimental setup of this study was performed both with and without HILIC enrichment of glycopeptides preceding the LC-MS/MS analysis. The rationale of this approach is the option to identify and semi-quantify the presence of non-glycosylated peptides for specific glycosylation sites and thereby target the macroheterogeneity of glycosylation sites. For the merged HILIC and non-HILIC datasets (Fig. 1*F*), the PCA plot (Fig. S6*A*) recapitulated the patient and control group separation of the previous HILIC-only dataset (Fig. 2*A*). Also, for the merged dataset, the glycan-only volcano plot, where the *NoGlycan* “glycoform” was included, a significant increase of non-glycosylated peptides was noted for TF and hemopexin (HPX, Fig. S6*B*). In addition, for the glycan-only volcano plot, the general increase of NoGlycan for PMM2-CDG was evident (Fig. S6*C*).

**Figure 6:**
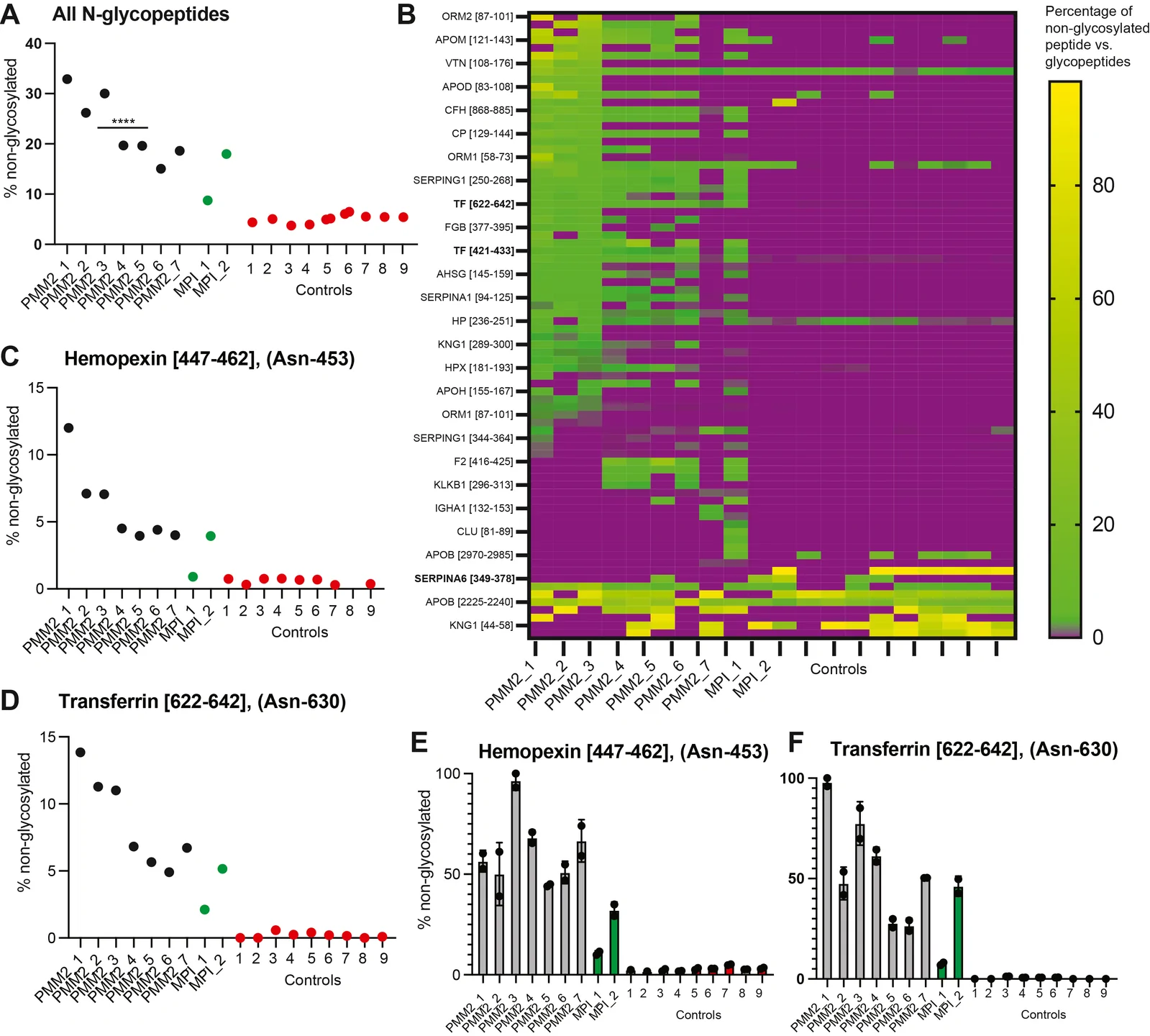
**Identification and relative abundance of non-glycosylated vs *N*-glycosylated peptides sharing the same peptide sequences.***A*, percentage of identified *N*-glycopeptide sequences that simultaneously were identified in non-glycosylated form. *B*, heatmap displaying the presence of non-glycosylated PSMs relative to the total number of PSMs and GPSMs for peptide sequences that were also detected in glycosylated form, across PMM2-CDG, MPI-CDG and control samples. Only entries where non-glycosylated peptides were identified in at least three of the PMM2-CDG samples are included (for the two MPI-CDG samples, one instance of non-glycosylated peptide is included). The entries for TF are shown in *bold*. *C*, example heatmap entries of hemopexin (HPX, Asn-453) and (*D*) transferrin (TF, Asn-630). See also Table S3. *E*, relative precursor ion intensities for the Asn-453 containing non-glycosylated peptide vs all identified (glyco)peptides from HPX using the non-HILIC datasets; and (*F*) the same for the Asn-620 non-glycosylated peptide from TF. See also Figure S7. The error bars show the standard deviation of double injections.

For the combined HILIC and non-HILIC datasets, a total of 233 *N*-glycan sites were identified in the PMM2-CDG sample 1, and 75 of these were also found to be non-glycosylated. Thus, 32.2% (75/233) of the *N*-glycan sites were identified to be partly unoccupied in this sample, whereas the other CDG patient samples showed less presence of non-glycosylated sites but typically higher than the controls (Fig. 6*A*). Importantly, all PMM2-CDG patient samples analyzed (n = 7) showed statistically significantly elevated levels of all non-glycosylated *N*-glycopeptides compared to the controls (*p* < 0.0001) (Fig. 6*A*). The levels were quite variable among the different patients but again divided into two groups, with PMM2-CDG patients 1 to 3 in one group and PMM2-CDG patients 4 to 7 in another group, and they were both clearly separated from the controls. To further characterize the relation of glycosylated vs. non-glycosylated peptides, the non-glycosylated peptides that were identified at least 3 times across the samples were used in a heatmap for a site-specific analysis (Fig. 6*B*). The raw data underlying the heatmap are provided in Table S3. The percentage of non-glycosylated PSMs relative to all GPSMs and PSMs for identified glycopeptide sequences varied substantially among patients. However, as a group, the mean values of non-glycosylated sites were for ∼50 glycopeptides increased for the PMM2-CDG and MPI-CDG patients compared to the controls, as further illustrated for HPX (Asn-453) and TF (Asn-630) and further glycopeptide entries (Figs. 6, *C*, *D* and S7).

In particular, non-glycosylated peptides covering both *N*-glycosylation sites of TF were identified in the CDG samples but scarcely in the controls (Figs. 6*D*, and S7). This result is in line with loss of one or two of the normally dominating disialo complex biantennary glycans, HexNAc(4)Hex(5)Neu5Ac(2), of serum TF, consistent with the lack of two or four sialic acids commonly used in the TF diagnostics of these CDGs. Notably, this major glycoform of the TF *N*-glycopeptides was not identified in the PCA as contributing to the separation between the PMM2-CDG and control samples (Table S2). As an additional assessment of relative abundances, using only non-HILIC data, we calculated the precursor ion intensity of selected non-glycosylated peptides relative to the summed intensity of all identified peptides and glycopeptides from the same glycoprotein (Figs. 6, *E*, *F* and S7). This analysis avoided direct comparison of signal intensities between HILIC-enriched and non-HILIC acquisitions and provided a protein-normalized estimate of the relative abundance of non-glycosylated peptides. The intensity-based results largely recapitulated the spectral count analysis, supporting increased detection of non-glycosylated peptides in the PMM2-and MPI-CDG samples.

Even though the relative abundance of non-glycosylated peptides was dominant for approximately 50 peptides in the PMM2-CDG samples, some non-glycosylated peptides were indeed found at similar levels in both PMM2-CDG and control samples, showing protein-specific variations in *N*-glycosylation occupancy (39). The *N*-glycopeptides with similar occupancy between the PMM2/MPI-CDG and controls are represented by the rows starting with the complement factor H (entry SERPINA6 [349–378]) in the heatmap (Fig. 6*B*). A complete list of the 80 identified non-glycosylated peptides, along with their corresponding protein names, are presented in Table S3.

### Increased presence of mono-glycosylated peptides containing two NXS/T motifs

**Figure 7:**
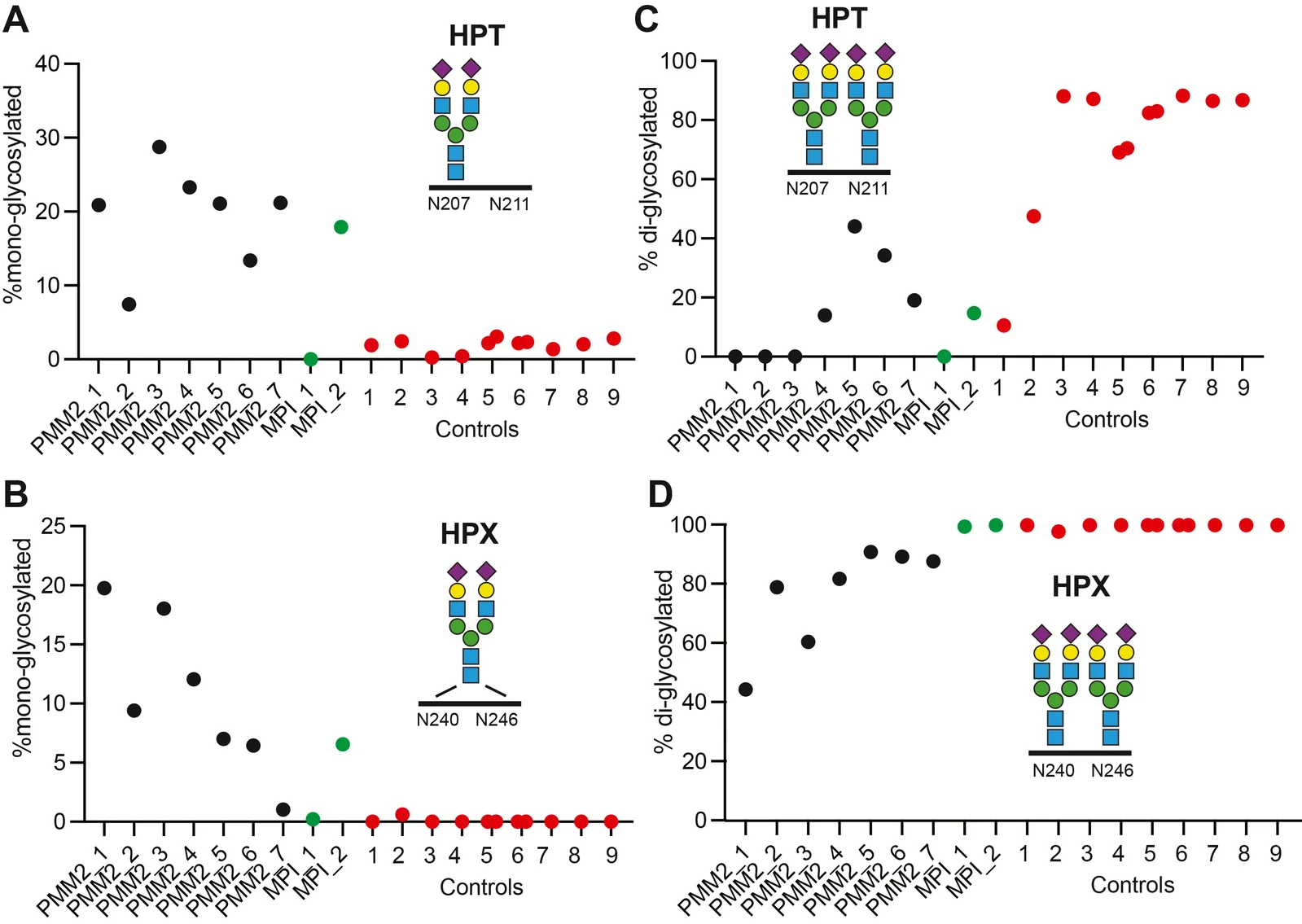
**Identification of mono-glycosylated peptides and doubly-glycosylated peptides with dual *N*-glycosylation sites.***A*, relative percentages of GPSMs of mono-glycosylated peptides containing the Asn-207 and Asn-211 glycosylation sites from haptoglobin (HPT) *versus* all GPSMs identified from HPT in the same individual samples (including all glycoforms, not only the displayed disialo complex biantennary glycan); and (*B*) relative percentage of GPSMs of mono-glycosylated glycopeptides for the glycopeptide containing the Asn-240 and Asn-246 glycosylation sites from hemopexin (HPX). *C*, relative percentages of GPSMs of the doubly-glycosylated peptides containing the Asn-207 and Asn-211 glycosylation sites from haptoglobin (HPT) vs all identified glycopeptides with the same peptide sequence; and (*D*) the same for doubly-glycosylated peptides containing the Asn-240 and Asn-246 glycosylation sites from hemopexin (HPX).

In addition to the increased occurrence of non-glycosylated peptides in PMM2-CDG patients, mono-glycosylated peptides containing two NXS/T N-glycosylation consensus motifs were observed in the PMM2-CDG samples, another sign of hypoglycosylation. Specifically, mono-glycosylated peptides containing the established Asn-207 and Asn-211 glycosylation sites of haptoglobin (HPT, Fig. 7*A*) and the Asn-240 and Asn-246 glycosylation sites of HPX (Fig. 7*B*) were elevated in the PMM2-CDG samples as compared to controls. Correspondingly, the *N*-glycopeptides of di-glycosylated HPT (Fig. 7*C*) and HPX (Figs. 7*D* and S8) were consistently more common in the healthy individuals. Another example of a mono-glycosylated sequence with dual NXS/T motifs was the M3-M4 paucimannose-like glycopeptides from IGHM, described above, which included two NXS/T motifs at Asn-279 and Asn-286 (Fig. 4*D*). Finally, a non-glycosylated peptide containing both the Asn-207 and Asn-211 glycosylation sites from HPT was identified in three of the PMM2-CDG samples (Fig. S9), and a mono-glycosylated peptide containing the NXS/T motif at Asn-183 and Asn-193 from apolipoprotein H (APOH) was identified in five of the PMM2-CDG samples but not in the MPI-CDG or in any of the control samples.

## Discussion

We set out to determine whether a discovery-driven semiquantitative plasma/serum glycoproteomic workflow could assist in identifying site-specific biomarkers for the most prevalent Type I congenital disorders of glycosylation (CDG), *that is,* PMM2-CDG. In unfractionated trypsin digests, analyzed with and without ZIC-HILIC, we observed a coherent two-part glycan signature composed of i) selective enrichment of paucimannose-like glycoforms (M3–M5) at specific glycosites, notably in C3 at Asn-85 with the GlcNAc(2)Man(4) glycoform (M4); in A2M at Asn-869 with the GlcNAc(2)Man(3) glycoform (M3); and in C4 at Asn-226 with the GlcNAc(2)Man(5) glycoform (M5); and ii) general increase in relative abundance and presence of peptides bearing unoccupied NXS/T *N-*glycosylation consensus motifs, including TF and dual-site peptides from HPT (Asn-207/Asn-211) and HPX (Asn-240/Asn-246). Several of these markers separated the patient cases from the controls, indicating that clinically informative signals are to be found in site-specific glycoforms and site occupancy rather than in gross shifts of the total plasma glycome. These observations align with and extend recent reports emphasizing the value of site-specific plasma glycoproteomics as biomarkers for CDGs (27, 28, 30).

**Figure 8:**
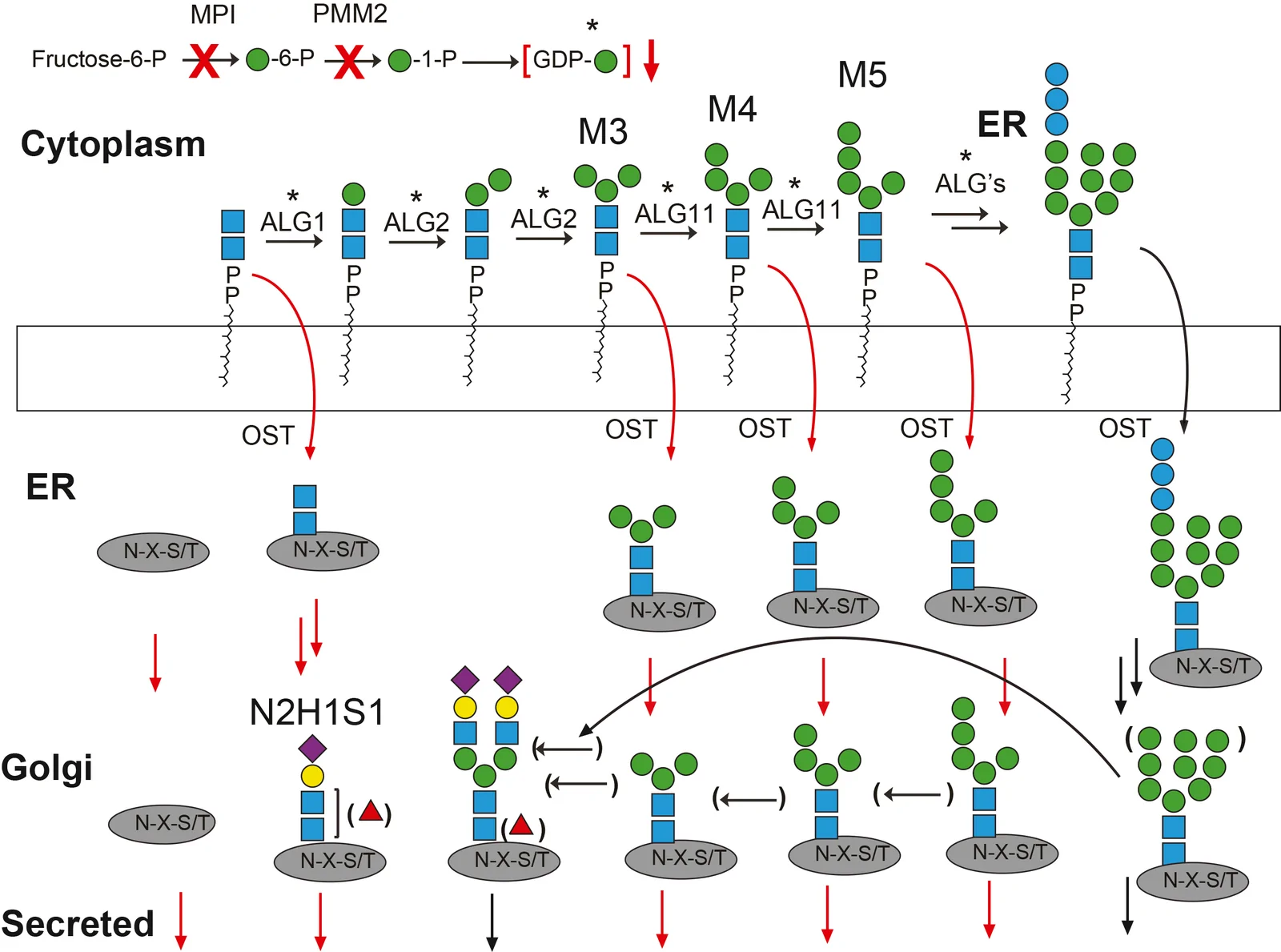
**Biosynthesis of GDP-Man and its incorporation into *N*-glycans.** Schematic overview of GDP-mannose production and lipid-linked oligosaccharide (LLO)-assembly in the cytoplasm, ER and Golgi. *Red arrows* indicate aberrant steps suggested to occur under conditions of GDP-mannose limitation (∗), based on the present findings, whereas *black arrows* indicate canonical biosynthetic steps in *N*-glycoprotein production. The general pathway layout is adapted from previous PMM2-and ALG1-CDG studies (26, 37) with modifications reflecting observations from this study.

PMM2 catalyzes the reversible interconversion of mannose-6-phosphate (M6P) and mannose-1-phosphate (M1P), whereas MPI generates M6P from fructose-6-phosphate, thereby supplying substrate for the PMM2-catalyzed reaction. M1P is subsequently converted to GDP-mannose, which is essential for the mannosylation of dolichol-phosphate–GlcNAc_2_, the lipid-linked oligosaccharide (LLO) precursor required for *N*-glycosylation (40). In the ER, multiple mannosyltransferases (*e.g.*, ALG1, ALG2, and ALG11) sequentially transfer mannose residues from GDP-mannose to the growing LLO precursor. Deficiencies in PMM2 (PMM2-CDG, CDG-Ia; OMIM #212065) and MPI (MPI-CDG, CDG-Ib; OMIM #602579) impair GDP-mannose production, resulting in insufficient LLO biosynthesis and transfer. Consequently, proteins that normally carry *N*-glycans become hypoglycosylated (Fig. 8). Although complex sialylated biantennary *N*-glycans dominate in plasma/serum of both patients and controls, we could show that a pronounced excess of M3/M4 in PMM2-/MPI-CDG distinguishes the patient cohorts from the controls (Fig. 3). This may theoretically be explained by the possibility that immature, LLO-derived M3-M5 glycans could be transferred and retained on proteins predisposed to display oligomannose in the circulation (Fig. 8, see also Fig. S3). However, the M5 composition, which was commonly observed on C3 and A2M in controls, may also arise from trimming of larger oligomannose structures after transfer to protein, and the present LC-MS/MS data do not unambiguously distinguish LLO-derived from processing-derived M5. Thus, we interpret the PMM2-CDG-associated C4-M5 finding as a site-specific enrichment of the M5 composition on C4 rather than definitive evidence for a single biosynthetic origin.

Proteins that exhibit oligomannose glycans in circulation are typically those that bypass full maturation in the Golgi apparatus. This can occur because of steric constraints, rapid protein folding, or, under pathological conditions, increased release of immature glycoproteins (41, 42). Increased presence of M3 and M4 in plasma from PMM2-CDG and related type I CDGs has been reported in glycomic studies (25, 26), but these approaches do not provide the protein- and site-specific information obtained here. Also, paucimannose glycans have been shown to be enriched in many cancer forms (36). Interestingly, C3 *N-*glycoprofiling reveals oligomannose plasma glycans that serve as early biomarkers of type 1 diabetes mellitus (21). In addition, under activated sugar donor limitation, fewer NXS/T motifs are glycosylated (39) and in line with this, we found a widespread reduction in *N*-glycosylation site occupancy of plasma glycoproteins. Thus, our findings align with hypoglycosylation, where increased presence of non-glycosylated peptides and mono-glycosylated peptides with dual NXS/T motifs occurs in CDG patients; alongside the selective increases of M3–M5 at approximately eight identifiable sites where oligomannose persists naturally, while the broad repertoire of complex-type glycans remains similar in patients and controls.

We also detected the aberrant N2H1S1 glycan, previously proposed as a biomarker for ALG1-CDG, in both the PMM2-and MPI-CDG patients (26, 37, 43). This observation is consistent with a diminished influx of GDP-mannose into the LLO biosynthesis pathway within the ER compartment (Fig. 8). Bengtson *et. al*. also detected N2H1S1 on TF in approximately half of their PMM2-CDG patients, albeit at significantly lower levels than in ALG1-CDG patients, and it was absent in MPI-CDG cases (37). This ALG1-CDG biomarker was interpreted as the tetrasaccharide GlcNAcGlcNAcGalNeu5Ac, and the authors suggested the mechanism summarized in Figure 8. In ALG1-deficient cells, dolichol-diphosphate-GlcNAcGlcNAc is produced, and this intermediate is normally the substrate for ALG1-mediated mannosylation. However, due to the enzyme deficiency, the dolichol-diphosphate-GlcNAcGlcNAc is translocated into the ER lumen, where the dolichol-diphosphate-linked GlcNAcGlcNAc disaccharide is transferred to nascent proteins by the oligosaccharyltransferase. As the glycoproteins move into the Golgi, the protein-bound GlcNAcGlcNAc template acts as an acceptor for galactosylation. The GlcNAcGlcNAcGal structure is subsequently extended by the addition of Neu5Ac by a sialyltransferase, thereby completing the biosynthesis of this tetrasaccharide. In addition, the fucosylated N2H1S1 composition has previously been reported as a released N-glycan in ALG1-CDG, with the fucose position assigned to the outer GlcNAc (25). Our data extend these observations by detecting N2H1F1S1 on specific glycopeptides, while annotating the fucose position as unresolved because of possible fucose migration.

Our glycoproteomic workflow makes use of a short 12 s exclusion time to balance repeated fragmentation of glycopeptide precursors with broader precursor coverage. Without dynamic exclusion, highly abundant peptides and dominant glycoforms would be repeatedly selected for MS/MS, reducing the chance of detecting low-abundance glycopeptides. This was particularly relevant for these PMM2-CDG and MPI-CDG samples, where diagnostically informative glycopeptides in the form of the N-tetrasaccharide glycopeptides were identified at relatively low intensity. The exclusion time was therefore kept short enough to allow repeated fragmentation of glycopeptides across the chromatographic peak, to support relative quantification using spectral counts while still enabling the detection of less abundant species. Accordingly, although the total number of unique glycopeptides was lower than in plasma glycoproteomics studies using more elaborate workflows (32), the spectra underlying the reported markers were of high quality and yielded strong identification scores.

In particular, the simultaneous assessment of non-glycosylated peptides and their corresponding glycosylated forms is challenging to address using intensity-based approaches but was enabled herein by merging the PSMs/GPSMs from HILIC and non-HILIC datasets. This approach should be interpreted as qualitative and semi-quantitative evidence of reduced site occupancy in PMM2-CDG and MPI-CDG, rather than as absolute occupancy measurements. Importantly, this analysis is not based on small differences in a few peptide signals, but on recurrent detection of non-glycosylated peptides across several plasma glycoproteins, as visualized in the heatmap (Fig. 7). To reduce the influence of differences between HILIC-enriched and non-HILIC acquisitions, we also evaluated precursor ion intensities of selected non-glycosylated peptides, within the non-HILIC datasets alone, in relation to the summed intensities of all detected peptides and glycopeptides from the same glycoprotein. This provided a protein-normalized estimate of the relative abundance of non-glycosylated peptides and largely recapitulated the spectral count-based results. Although ionization efficiency, peptide detectability, and protein abundance may still influence the measurements, the repeated identification of such peptides in CDG patient samples provides strong complementary evidence for under-occupied glycosylation sites.

Since a major part of our glycoproteomic quantitation methodology was based on GPSM identification frequency rather than peak intensities, it provided a straightforward workflow where complications related to chromatographic peak picking and integration were avoided. Assessment of absolute protein or peptide concentrations was not necessary, as relative quantification was carried out within each sample and subsequently compared across groups. Thus, our strongest conclusions were whether relative glycoform patterns differed consistently between CDG and control samples. Moreover, our workflow supports a broad discovery setting without requiring prior selection of specific glycopeptides. *O*-glycopeptides were not included in the present study, as the primary focus was on PMM2-CDG, disorders that predominantly affect *N*-glycosylation. Consequently, the HILIC enrichment used is optimized for *N*-glycopeptides and is less effective for the shorter *O*-glycans. Dedicated *O*-glycoproteomic approaches may be relevant in future CDG studies. Furthermore, our methodology may also be applicable to patient samples from other CDG subtypes, including type II CDG conditions, and could facilitate further identification and comparative assessment of candidate biomarkers linked to CDG pathophysiology.

Recent findings highlight enhanced diagnostic and analytical potential of site-specific plasma glycoproteomics in the context of CDGs (28, 30, 44). This approach allows for a more nuanced understanding of glycosylation abnormalities at the molecular level, offering promising avenues for biomarker discovery, patient stratification, and therapeutic monitoring (30). In contrast to our methodological design, Garapati *et al.* (30) employed a stepwise pipeline for PMM2-CDG plasma samples (n = 7 in the discovery set, overall n = 36) using tandem mass-tag (TMT) multiplexing, glycopeptide enrichment, and prefractionation to enable between-sample reporter ion quantification, followed by a targeted LC-MS/MS approach (30). While TMT is well suited for groupwise comparisons, it may obscure site-specific glycoform distributions due to co-isolation of multiple (glyco)peptides in complex mixtures, potentially leading to ratio compression. In contrast, our approach enables the assessment of glycan microheterogeneity and site occupancy within a single analytical setup with moderate sample handling, which may be advantageous in a clinical context (16).

Despite the different methodological approaches, Garapati *et al.* and our study converge on complement proteins as promising biomarker candidates. They identified elevated M5 glycan at Asn-226 of C4 in PMM2-CDG compared to controls. Importantly, this biomarker remained detectable even when serum TF glycoform analysis was normal and decreased during epalrestat treatment in one individual (30). These findings represent a significant advance in biomarker discovery for PMM2-CDG. We also observed elevated M5 glycan at Asn-226 of C4 in PMM2-/MPI-CDG and extended the biomarker repertoire with M4 at Asn-85 of C3; and M3 as well as M4 at Asn-869 of A2M (Fig. 4). We also identified mono-glycosylated HPT peptides in PMM2-CDG, consistent with site-specific hypoglycosylation (30), and extend the potential biomarker repertoire with mono-glycosylated HPX, APOH and IGHM peptides (Fig. 7). Together, these corroborating findings under different analytical conditions strengthen the hypothesis that selected complement-derived oligomannose glycopeptides plus mono-glycosylation occupancy of dual *N*-glycosites capture core molecular features of Type I CDG.

Notably, the highest proportion of the M3/M4 glycan structures in our study was found in infants, which may reflect an increased susceptibility to glycosylation defects compared to older children and adults. The youngest PMM2-CDG infants (<1 year) showed the highest M4 percentages (>3% of all GPSMs), with older individuals clustering at lower but still elevated levels (≈0.8–1.5%). Controls were near or below the detection limit (<0.1%). It is well established, particularly in PMM2-CDG, that disease progression tends to stabilise with age, resulting in a more consistent phenotype over time (45). However, the potential association between the relative abundance of M3/M4 glycoforms at specific glycosites and genotype and/or clinical phenotype remains to be elucidated.

Given the limited number of patients included in our study, our findings require validation in a larger cohort of PMM2-CDG patients. Furthermore, the inter-individual heterogeneity in glycopeptide biomarker amplitudes might reflect the status of specific cell metabolic pathways influencing oligomannose retention, secretory residence times, enzyme access and activity, as well as other confounding person-related factors (genotype, age, inflammation, etc). Complement and other acute-phase proteins vary with inflammatory state, potentially shifting glycoprotein abundances. Our markers mitigate these interferences by emphasizing glycoform distributions and site occupancy, ratios less sensitive to concentration changes. However, validation in inflammatory and liver-disease cohorts will be important for further studies of biomarker specificity.

This study has some limitations such as cohort size, as discussed earlier, age of subjects and matrix heterogeneity (serum and plasma), which constrain the precision of performance estimates and reference ranges. In addition, the spectral count-based semi-quantification applied here does not necessarily reflect absolute or true relative abundances between samples. Nevertheless, significant and consistent differences were observed between patients and controls, particularly for features that were sparsely detected in controls but consistently observed in PMM2-and MPI-CDG samples. Furthermore, our glycoproteomic platform is not designed for use in a clinical diagnostic workflow. Accordingly, we employed precursor ion intensity measurements as a complementary analytical approach for selected non-glycosylated peptides in the non-HILIC datasets and for M3–M5 oligomannose-like glycopeptides in the HILIC datasets. These analyses largely recapitulated the spectral count-based results and may represent a step toward the development of a method that is more suitable for clinical diagnostic applications.

## Conclusion

The integrated glycoproteomic workflow presented here captures the two fundamental biochemical consequences of Type I CDG, reduced site occupancy and significant presence of M3-M5 paucimannose-like glycosylation at selected sites, in the same experiment. These findings replicate and extend recent observations (notably C4-M5) and add new, independent potential biomarkers (C3-M4, A2M-M3) suitable for translation to targeted assays. Also, many non-glycosylated peptides of *N*-glycopeptide sequences were identified in the PMM2-CDG samples. We anticipate that a compact, site-specific targeted mass spectrometry panel will complement serum TF analysis and genetics for future diagnosis and monitoring of PMM2-CDG.

## Future directions

Future study priorities include (i) Validation of candidate glycophenotypes with potential utility as biomarkers for PMM2-CDG in independent patient cohorts (ii) conversion of the biomarker set into a targeted LC-MS/MS assay with selected glycopeptides and non-glycosylated peptides; (iii) specification of age- and gender-specific cutoffs. Age-dependent differences in biomarker magnitude argue for age-stratified reference or continuous intervals (46); iv) evaluation of LC-MS/MS performance *versus* genotype and clinical phenotype; and v) evaluation of the usefulness of our presented glycoproteomic platform in patient samples exhibiting other CDG conditions, especially those with type II CDG profiles, to enhance the comprehensive identification and characterization of novel biomarkers associated with CDG pathophysiology.

## Experimental procedures

### Materials

Plasma and serum samples were obtained from nine patients with PMM2-and MPI-CDG diagnoses (Table 1), established from serum TF and genetic analyses. Samples were collected at the Clinical Chemistry Laboratory, Sahlgrenska University Hospital, Gothenburg, Sweden. Li-heparin plasma control samples (n = 9, Table S1) were obtained from the same laboratory after analysis of thyroid-stimulating hormone (TSH). Only plasma samples with normal levels of TSH were selected as controls. The control individuals were aged 3 months to 58 years, collected to match the ages of the CDG cases. Patient samples were analyzed in two separate batches, with control samples C5 and C6 included in both batches.

### Ethical consideration

This study was approved by the Swedish Ethical Review Authority (EPN 2023-06761-0) and specific informed consent was waived. This work was carried out in accordance with the code of ethics of the World Medical Association (Declaration of Helsinki).

### Sample preparation

Plasma/serum sample aliquots (6 μl) were added to digestion buffer (50 μl end volume) containing a final concentration of 0.1% sodium deoxycholate (SDOC), 50 mM triethylammonium bicarbonate (TEAB) and 10 mM dithiothreitol (DTT). The reduction was performed at 56 °C for 20 min, and then cysteine alkylation proceeded with 20 mM iodoacetamide (IAA) at room temperature for 30 min in the dark; and finally, a new portion of 10 mM DTT was added, and the quenching of IAA was done for 15 min. An additional portion of 35 μl TEAB (100 mM) was added, and the protein digestion was performed at 37 °C for 14 h with a trypsin/LysC mixture (Promega), at 50:1 substrate: enzyme ratio (approximating 0.4 mg protein in each sample) in an end volume of 100 μl. A second incubation was then done with trypsin (sequencing grade, Thermo Scientific) at 50:1 for 3.5 h at 37 °C.

After proteolysis the samples were subjected to HiPPR detergent removal resin bulk material (0.4 ml per sample, Thermo Scientific) according to instructions. The samples were then acidified with trifluoroacetic acid (TFA) to pH < 2 to precipitate remaining SDOC, centrifuged at 1000 × *g* for 3 min and the supernatants subjected to C18 spin columns (Thermo Scientific) according to instructions. The elution of peptides was done with 2 × 100 μl 40% acetonitrile in ultrapure water. Each sample was divided into a one-fourth fraction and a three-fourths fraction and dried in a vacuum centrifuge at room temperature. The three-fourths fractions were re-dissolved in 200 μl 80% acetonitrile/1% TFA and subjected to zvitterionic hydrophilic interaction liquid chromatography (Zic-HILIC) for the enrichment of glycopeptides (13) The in-house packed spin columns contained 80 mg Zic-HILIC particles (10 μm porous silica, 200 Å; Sequant/Merck) and the samples were applied and re-applied four times with centrifugations at 1000g. Each column was then washed once with 250 μl of 80% acetonitrile, 1% TFA. Enriched glycopeptides were eluted with 3 × 60 μl of 0.1% TFA followed by 60 μl of 25 mM TEAB and finally by 60 μl of 70% acetonitrile; and then dried by vacuum centrifugation at room temperature.

### LC-MS/MS analysis

For the LC-MS/MS analysis, the HILIC enriched glycopeptide and the non-HILIC peptide samples were dissolved in 60 μl and 90 μl 3% acetonitrile, 0.1% TFA, respectively. The samples were analyzed on an Orbitrap Lumos Tribrid mass spectrometer (Thermo Scientific) interfaced with the Easy-nLC1200 chromatography system (Thermo Scientific). The ion-transfer tube temperature was 275 °C and the positive ion spray voltage was 2 kV. Analytes (2 μl injection volume for HILIC samples and 1 μl for non-HILIC samples) were trapped on an Acclaim Pepmap 100 C18 trap column (100 μm × 2 cm, particle size 5 μm, Thermo Fisher Scientific) and separated on an in-house packed analytical column (75 μm × 45 cm, particle size 3 μm, Reprosil-Pur C18, Dr Maisch), maintained in a temperature-control sleeve at 50 °C. The mobile phases were solvent A (0.2% formic acid in water) and solvent B (80% acetonitrile, 0.2% formic acid in water); and the flow rate was 300 nl/min. The elution gradient started with 5% B, going to 8% B over 5 min, followed by an increase to 35% B over 68 min, to 45% B over 5 min, to 100% B over 2 min and then remained at 100% B for 15 min.

The MS^1^ scans were acquired at 120,000 resolution with an *m/z* 600 to 2200 range (HILIC samples) or *m/z* 400 to 2000 (non-HILIC samples), and the most abundant 2 to 7 charged precursor ions were selected for fragmentation using higher-energy collision-induced dissociation (HCD) with a duty cycle of 3 s and an isolation window of 3 m/z units (3 Th). The HILIC samples from patients were analyzed with triplicate injections, where the first injection was analysed with 20% and 30% normalized collision energy (NCE) in the *m/z* 109 to 2400 range (to include the diagnostic His immonium ion at *m/z* 110.07). The second and third injections were analyzed with 38% NCE in *m/z* 200 to 2000 range. For the non-HILIC samples, 30% NCE was used in the *m/z* 109 to 2000 range. The exclusion time was 12 s, precursor intensity threshold for MS^2^ was 5 × 10^4^, MS^2^ resolution was 30,000, and the maximum injection time was 90 ms. Two blank injections with isopropanol:acetonitrile:water (1:1:1) followed by solvent A were performed in between samples from different individuals to minimize and monitor any carry-over of analytes.

### Glycoproteomic identification of glycopeptides

Data analysis was performed with Proteome Discoverer version 3.0 (Thermo Fisher Scientific) using the Byonic search engine (v. 4.3.4, Protein Metrics) towards the human UniProt database (2023_02; 20,422 entries). The *N*-glycan modifications were set to 204 compositions ranging from *HexNAc*(1) to *HexNAc*(7)*Hex*(8)*Fuc*(1)*NeuAc*(4) (Table S4). One glycan was allowed per peptide (*common1* setting), but additional analyses were performed using two allowed glycans and the *common2* setting. Additional dynamic modifications were Met oxidation and loss of NH_2_ from N-terminal Gln and Cys, where one of these was allowed per peptide (*rare1* setting); and finally fixed carbamidomethyl group of Cys. Allowed peptide cleavage sites were after Lysine (K) and Arginine (R), including two missed cleavages, and the mass accuracy limit was 5 ppm for precursor ions and 20 ppm for MS^2^ ions.

The false discovery rate was set to 1%, and an additional protein-aware posterior error probability (PEP2D) score of <0.001 was used in the initial filtering of GPSMs and PSMs. Selected glycopeptide MS^2^ spectra were manually verified to contain peaks confirming the matched peptide (Y0), peptide + HexNAc (Y1) and peptide + HexNAcFuc (core-fucosylated *N*-glycans) ions. Also, the proper set of diagnostic saccharide oxonium ions had to be present, for instance, presence of *m/z* 274.09 for compositions including Neu5Ac and *m/z* 163.06 at >20% relative abundance (at 20–30% NCEs) for compositions including oligomannose *N*-glycosylation.

### Data and statistical analysis

For the GPSM-based relative quantification analyses, PCA-plots and volcano plots, the two replicate measurements of the HILIC samples made at 38% NCE were summed and used. The identification of non-glycosylated peptides of corresponding *N*-glycopeptides also detected in the same sample was assessed by adding the GPSMs from the two HILIC runs (NCE 38%), to the PSMs and GPSMs of the two non-HILIC runs (NCE 30%) and then filtering for the simultaneous presence of GPSMs and non-glycosylated PSMs sharing the same peptide sequence. The analysis strategy is summarized in Figure 1.

For the Minora MS1 precursor ion intensity quantifications of the HILIC samples, the three measurements (one at 20%/30% NCE and two at 38% NCE), which were identical for the relevant MS1 part, were used for PMM2 and MPI samples, and the two available measurements at 38% NCE were used for the control samples. After filtering for glycopeptide (glycan composition contains *Hex*), the Peptide groups, Minora consensus features and PSM tables were imported into R-studio for processing. Data was summarized by aggregating spectrum counts at the glycosylation site level (>1 GPSM identity threshold), protein level (>2 GPSM identity threshold), and at the glycan composition level. The resulting summary tables were exported to Excel spreadsheets for further analysis and visualization. Relative glycoform abundances were assayed by summing GPSMs for each glycan/glycopeptide and normalizing to the total GPSM signal or to selected glycoproteins/*N*-glycosylation sites within each sample, enabling comparisons and statistical analyses across samples.

Statistical analyses were performed in R (v4.5.0) and in GraphPad Prism (v10.0). The glycopeptide- and glycan-level datasets were imported and processed separately. An unpaired two-tailed *t* test was used to compare PMM2-CDG (n = 7) and control samples 2 to 8 (n = 7) in Prism. Significance was defined as *p* < 0.05. The relative spectrum counts of GPSMs for each sample were log2-transformed with a pseudo-count of one for zero values prior to the PCA and Volcano analyses. PCA was carried out using the Factominer package in R with unit variance scaling set to off. Group-wise differential testing between PMM2-CDG and control samples was performed using two-sided Welch’s t-tests applied to each glycopeptide or glycan independently. Effect sizes were calculated as log2 fold changes based on the mean log2-transformed relative abundances from each group, and plots were generated using ggplot2, displaying log2 fold change *versus* the negative log10 *p*-value. Raw *p*-values were reported without multiple-testing correction. Significance categories were assigned according to predefined thresholds for *p*-value and absolute log2 fold change.

## Data availability

Data is presented in the manuscript and its Supporting Information files. The MS proteomics data and Byonic viewer files have been deposited to the Proteome Xchange Consortium *via* the PRIDE (47) partner repository with the dataset identifier PXD080194. Spreadsheets with the GPSM datasets are available.

## Supporting information

This article contains supporting information.

## Conflict of interest

The authors declare that they have no conflicts of interest with the contents of this article.
