# Supplementary material for: Integrated glycoproteomics reveals site-specific N-glycosylation defects in phosphomannomutase two congenital disorder of glycosylation
Source: J Biol Chem. 2026 Aug 19;302(10):113469. doi: 10.1016/j.jbc.2026.113469 (PMC13597197; doi:10.1016/j.jbc.2026.113469)
Supplement: Supplementary file 1 [file mmc1.pdf]

Supporting Information:

Integrated glycoproteomics reveals site-specific N-glycosylation defects in phosphomannomutase 2 congenital disorder of glycosylation

Jonas Nilsson[1,2], Ekaterina Mirgorodskaya[1,2], Carina Sihlbom Wallem[1,2], Fredrik Noborn[3], Erik A Eklund[4,5], Göran Larson[3,6] and Maria Blomqvist[3,6]

[1]Proteomics Core Facility (PCF) and BioMS, Sahlgrenska Academy, University of Gothenburg, Gothenburg, Sweden.

[2]Glycoproteomics and MS Proteomics, Science for Life Laboratory (SciLifeLab), University of Gothenburg, Gothenburg, Sweden

[3]Department of Laboratory Medicine, Institute of Biomedicine, University of Gothenburg, Gothenburg, Sweden

[4]Section for Pediatrics, Department of Clinical Sciences, Lund University, Lund, Sweden

[5] VO Barnmedicin, Skåne University Hospital, Lund, Sweden

[6]Department of Clinical Chemistry, Sahlgrenska University Hospital, Gothenburg, Sweden

Corresponding author:

Maria Blomqvist, PhD, Department of Clinical Chemistry, Sahlgrenska University Hospital,

SE 413 45 Gothenburg, Sweden

E-mail: maria.k.blomqvist@vgregion.se, Phone: +46 73 867 8124

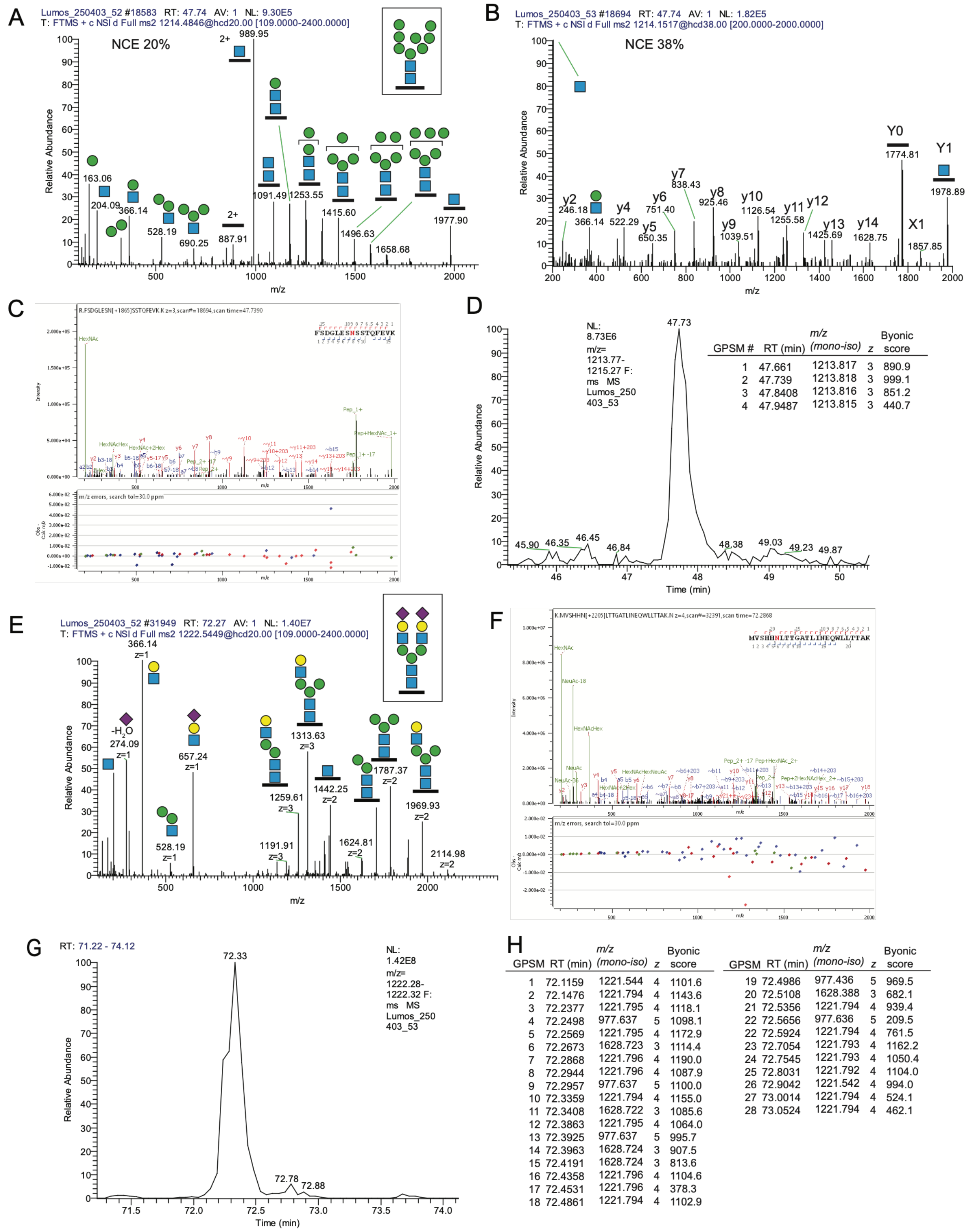

Supporting Fig. 1. LC-MS/MS of plasma N-glycopeptides (**A**) HCD-MS2 spectrum at 20% normalized collision energy (NCE) demonstrate the major presence of B-ions (oxonium ions) and glycosidic fragment ions with an intact peptide (Y-ions). The oligomannose N-glycan has GlcNAc(2)Man(9) composition and the peptide is FSDGLESNSSTQFEVK including the Asn-226 N-glycosite of complement 4-A and 4-B (C4). (**B**) The same precursor ion at 38% NCE demonstrating the dominant presence of peptide backbone fragmented y-ions and low presence of B-ions and glycosidic fragment ions. (**C**) The Byonic-annotated spectrum of the MS2 spectrum in panel **B**

showing annotation of a nearly complete b-and y-ion series. (**D**) Extracted ion chromatogram (XIC) demonstrating the elution profile and time points of the Byonic-identified glycopeptide spectrum matches (GPSMs) for the glycopeptide in panel **A**-**C**. (**E**) HCD-MS2 spectrum at 20% NCE for the disialo complex biantennary N-glycopeptide with the peptide MVSHHNLTTGATLINEQWLLTTAK including the Asn-184 N-glycosite of haptoglobin; and (**F**) the Byonic-annotated spectrum at 38% NCE again showing the dominant presence of b- and y-ions. (**G**) The XIC of the glycopeptide in panel **E** and **F**; and (**H**) the corresponding time points of the Byonic-identified GPSMs demonstrating 28 identifications during 1 min.

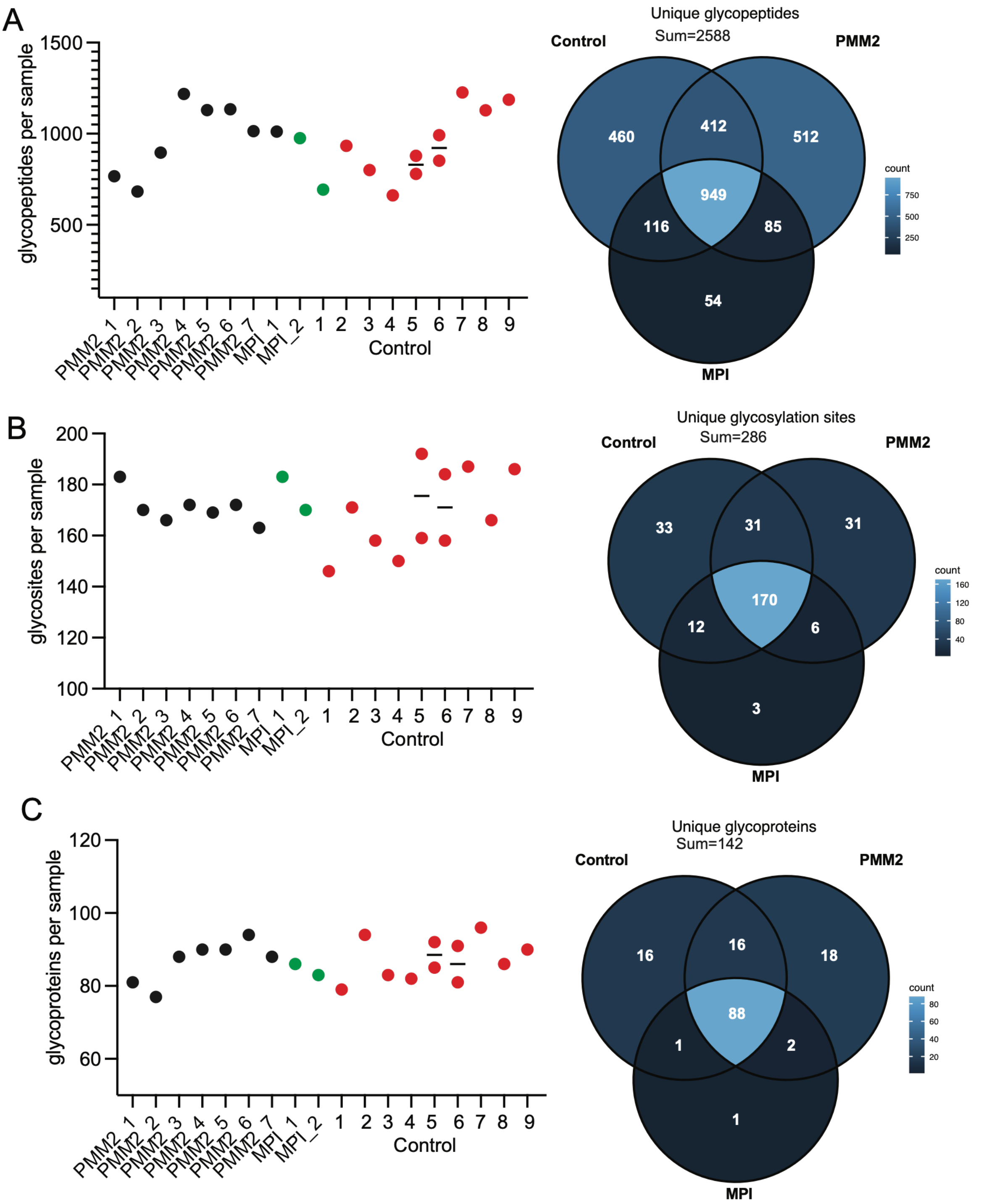

Supporting Fig. 2. (**A**) Total number of unique glycopeptides in the PMM2-CDG, MPI-CDG and control samples (left); and a Venn diagram summarizing the glycopeptides across the three sample groups (right). (**B**) The same for unique glycosites per samples. (**C**) The same for number of identified glycoproteins per sample. Control samples 5 and 6 were prepared and analyzed at two occasions.

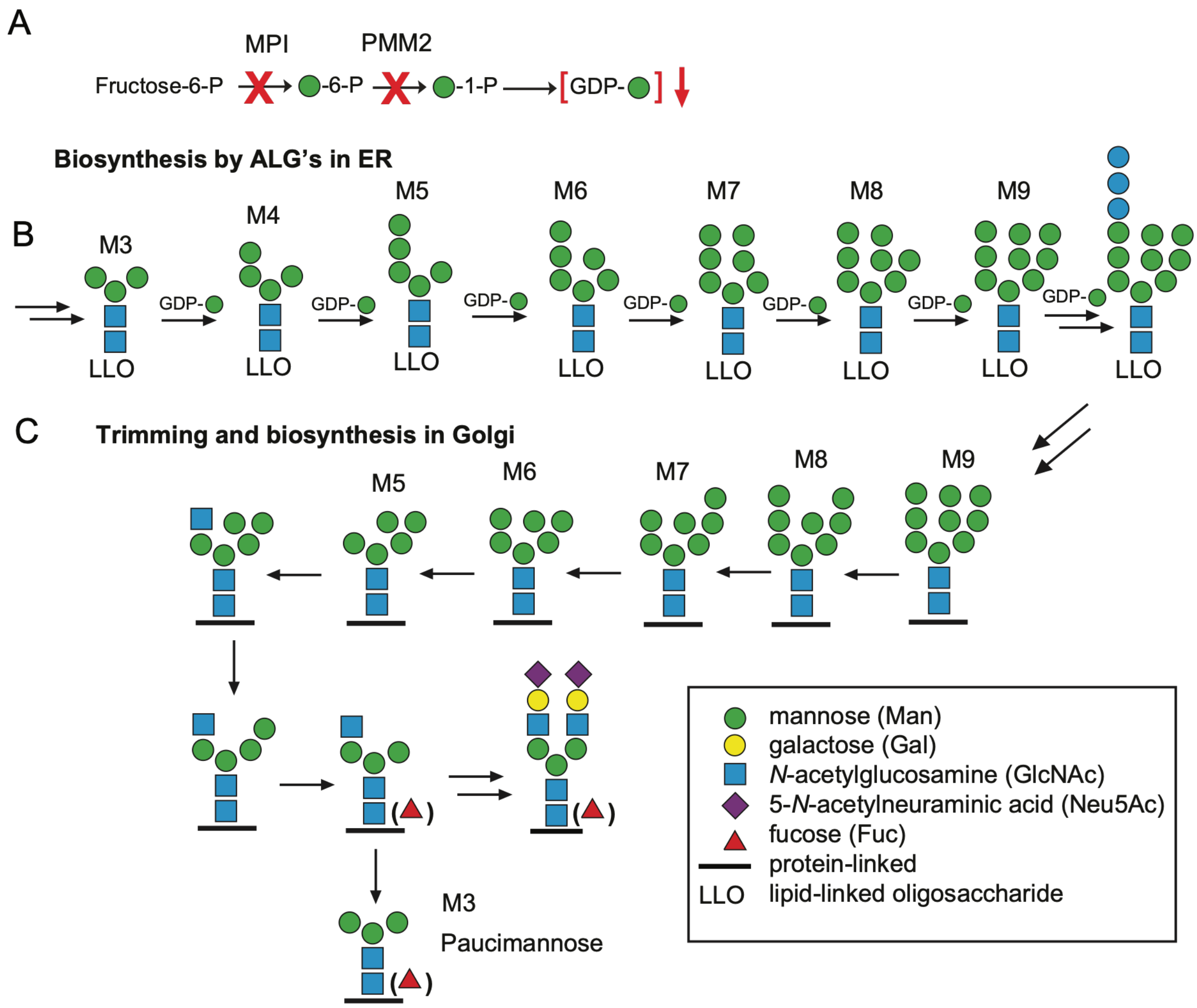

Supporting Fig. 3. Cellular biosynthesis of N-glycans. (**A**) Metabolic production of GDP-Man, which is suppressed in the MPI-CDG and PMM2-CDG conditions. (**B**) Oligomannose assembly (M3-M9), using GDP-Man, by the asparagine-linked glycosylation (ALG)-transferases in the endoplasmatic reticulum (ER). In this phase the glycan is a lipid-linked oligosaccharide (LLO), which then is transferred to Asn glycosylation sites. (**C**) Trimming and synthesis of the protein-linked oligomannose (M5-M9), paucimannose-like (M3) and complex type N-glycans. Note that the Man connectivity differ for LLO and protein-linked M5-M8, and that the M4 composition does not occur protein-linked. The figure is adapted from *Essentials in Glycobiology, chapter 9*.

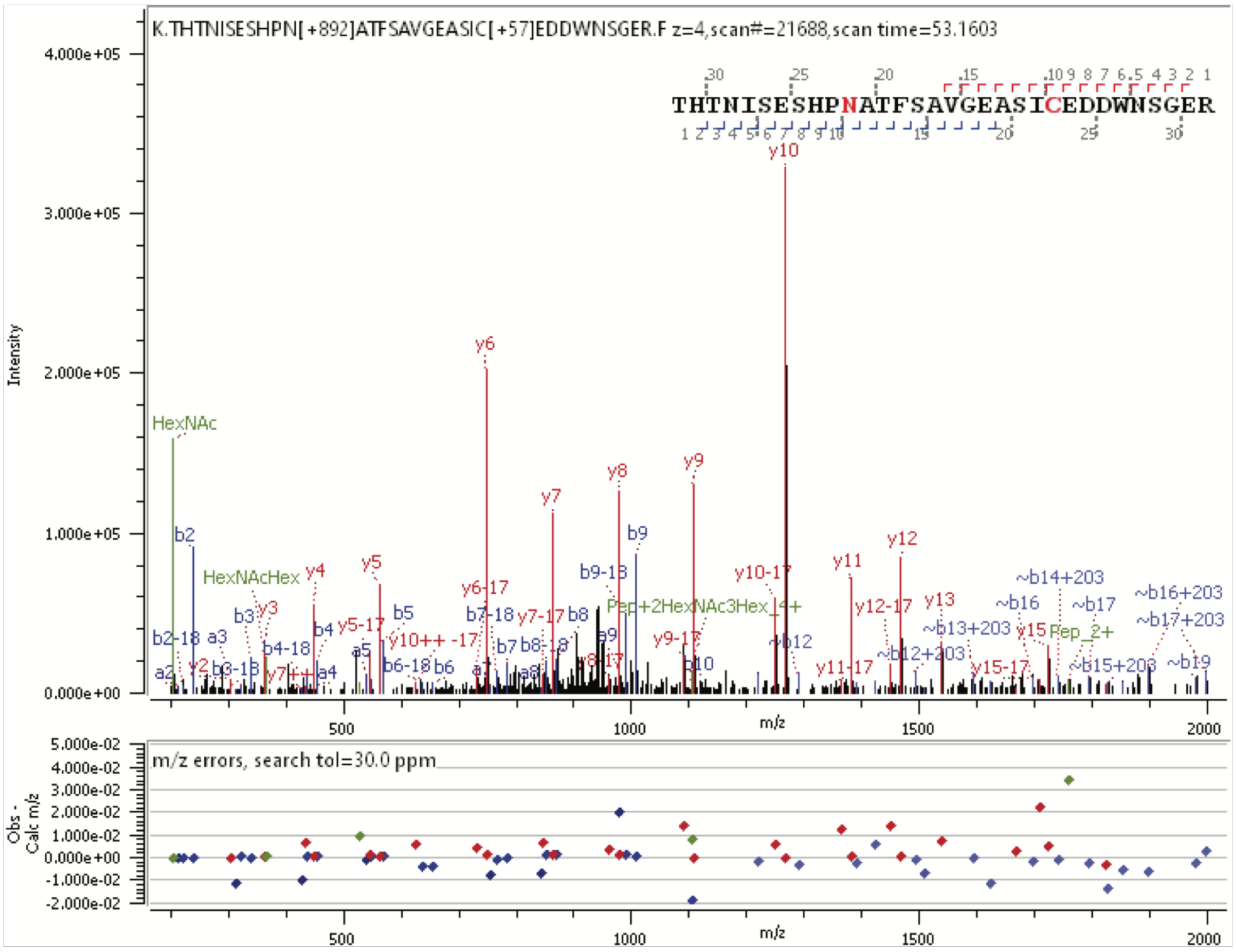

Supporting Fig. 4. Byonic-annotated MS2 spectrum of the M3 glycoform of a glycopeptide [amino acids 269-300] of immunoglobulin heavy constant mu (IGHM) with dual NXS/T motifs. Inspection of the annotated b- and y-ions revealed that all GPSMs showed the presence of non-glycosylated b-ions up to b9, including only Asn-279, whereas b11 and larger, including both sites, all included HexNAc (+203). This confirmed that Asn-279 served as the glycosylation site.

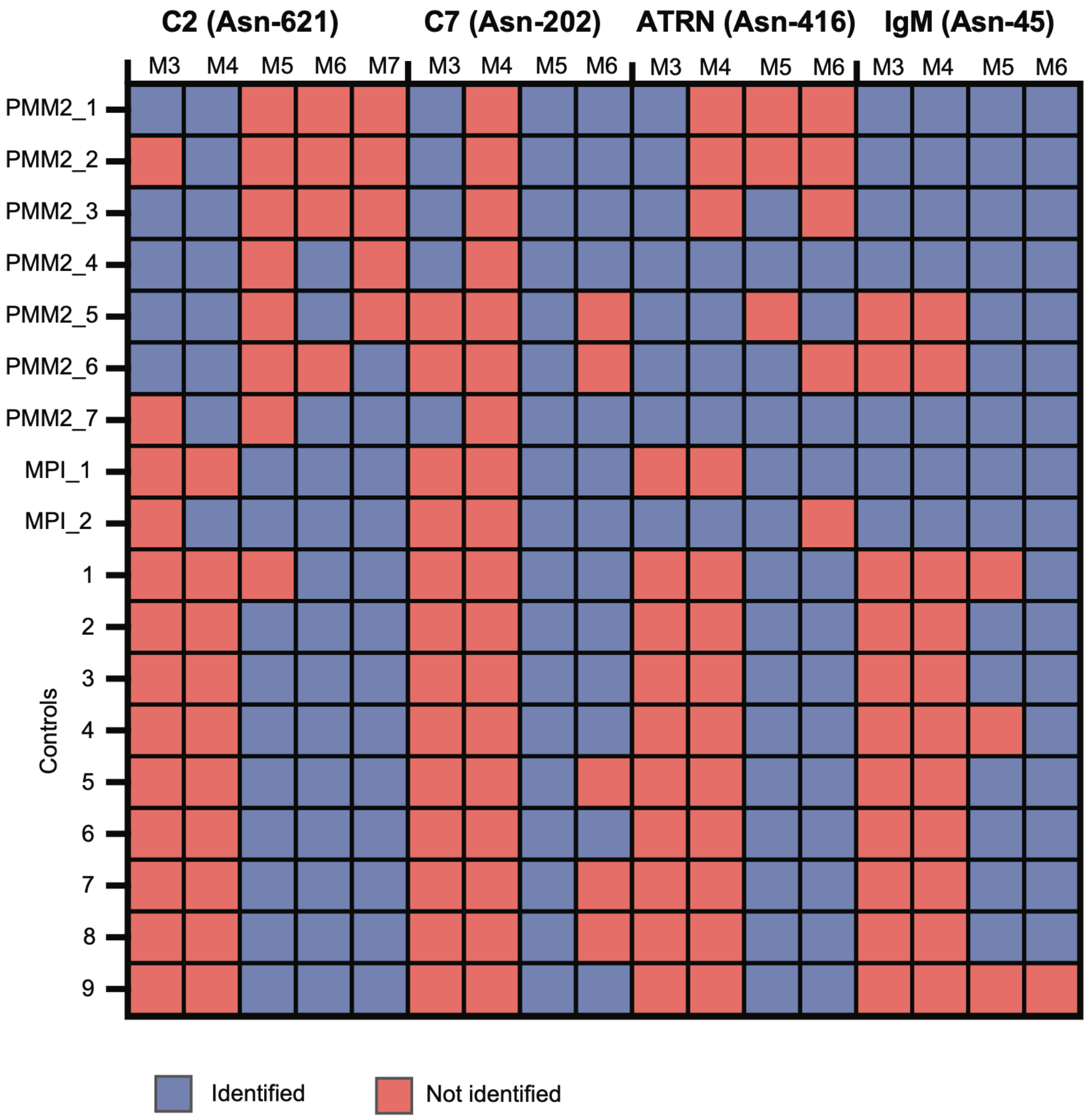

Supporting Fig. 5. Distribution of additional truncated oligomannose (M3-M7) glycoforms identified at C3, C7, ATRN and IGHM N-glycosylation sites in PMM2- and MPI-CDGs and in controls. The M3 and M4 glycoforms were identified for Asn-621 of complement C2, Asn-202 of complement C7, Asn-416 of attractin (ATRN) and Asn-45 of immunoglobulin heavy constant mu (IGHM) in the CDG samples. M3 of these four glycosylation sites was identified in a majority of the PMM2 samples but not in any of the control samples. M4 was similarly identified in a majority of the PMM2 samples but was totally absent in C7 of all patients and all control samples. Overall, PMM2 samples 5 and 6 showed a minor presence of M3 and M4 glycoforms. For the two MPI samples, only Asn-45 of IGHM was identified as carrying the M3 and M4 glycoforms.

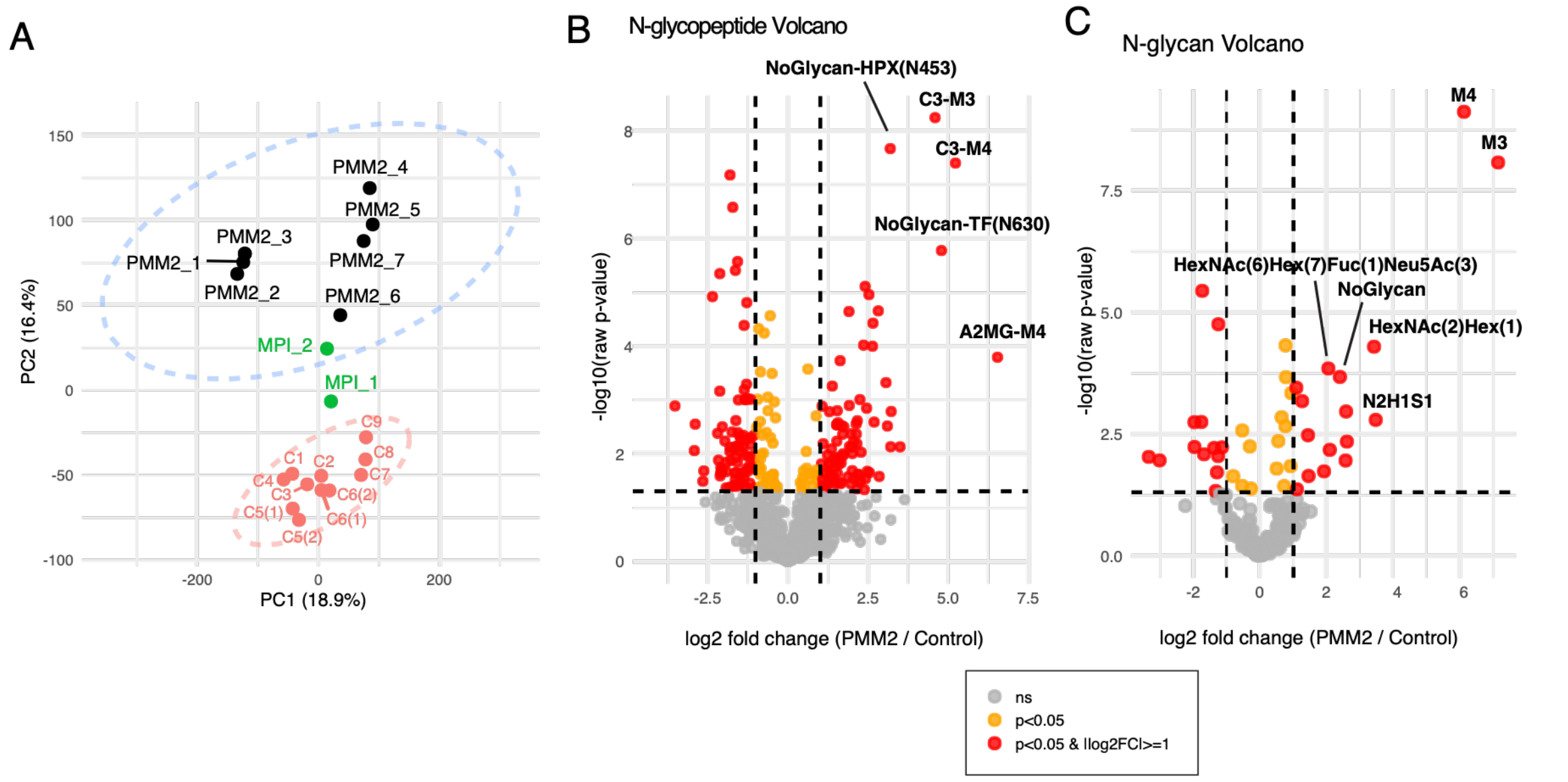

Supporting Fig. 6. Data analyses of the merged HILIC and non-HILIC datasets. (**A**) The PCA plot of the glycopeptides. (**B**) Volcano plot of the glycopeptide datasets. The glycoform "NoGlycan" is implemented in the analysis. (**C**) A Volcano plot of the glycomic profiles where the glycosylation site information is removed from the analysis. Two-sided Welch's t-tests were implemented. Control samples 5 and 6 were prepared and analyzed by LC-MS/MS at two different occasions.

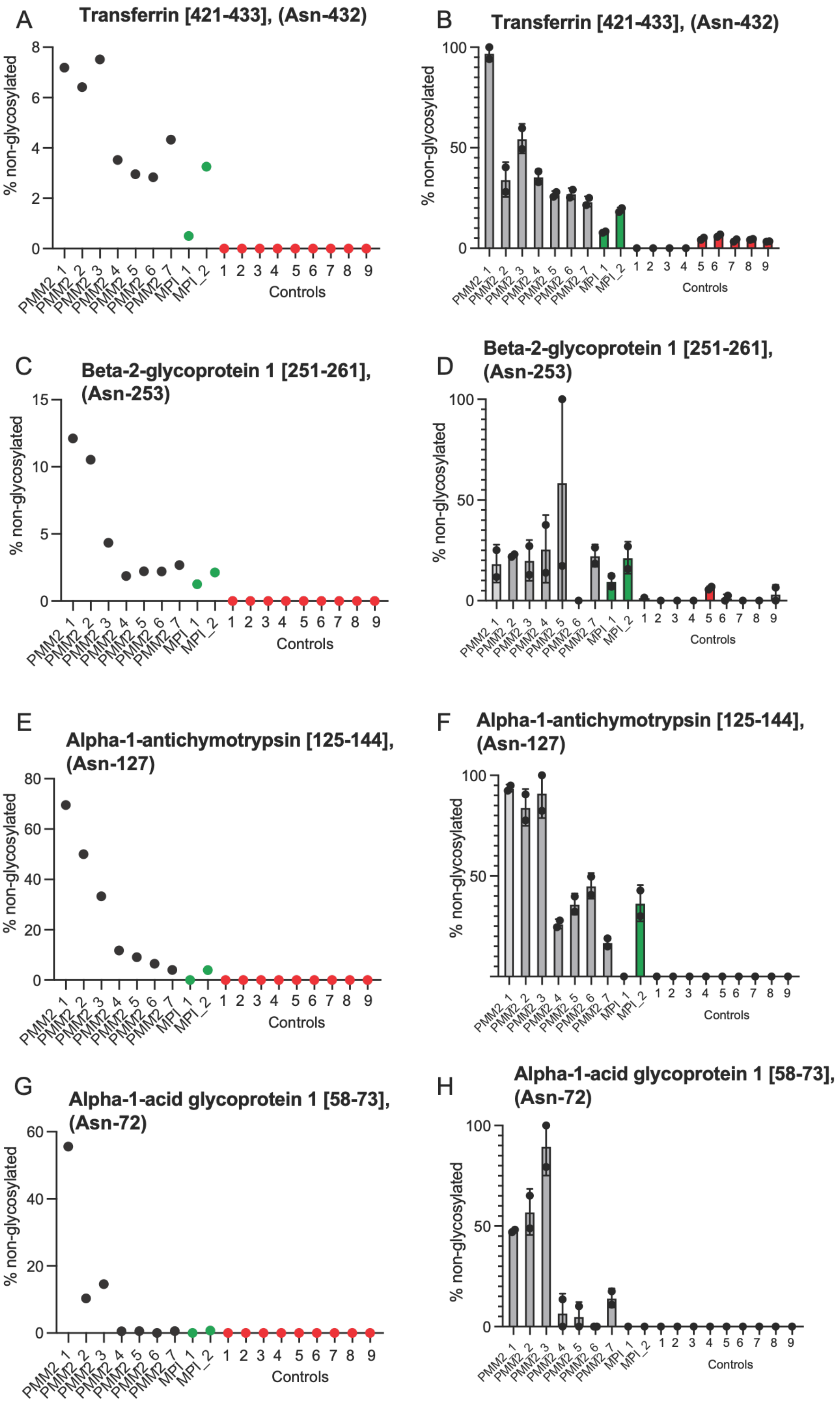

Supporting Fig. 7. Relative abundance of selected non-glycosylated N-glycosylation sites in PMM2- and PMI-CDG and control samples. The percentages were calculated from non-glycosylated peptide spectral matches (PSMs) of peptides containing a NXS/T consensus motif versus all PSMs and GPSMs from the same peptides (left panels); and precursor ion intensities of the same non-glycosylated peptides vs. all intensities from the same protein using only non-HILIC samples (right panels). The precursor ion intensity percentage values were normalized to 100% with the highest measured value in each series. The error bars show the standard deviation of double injections.

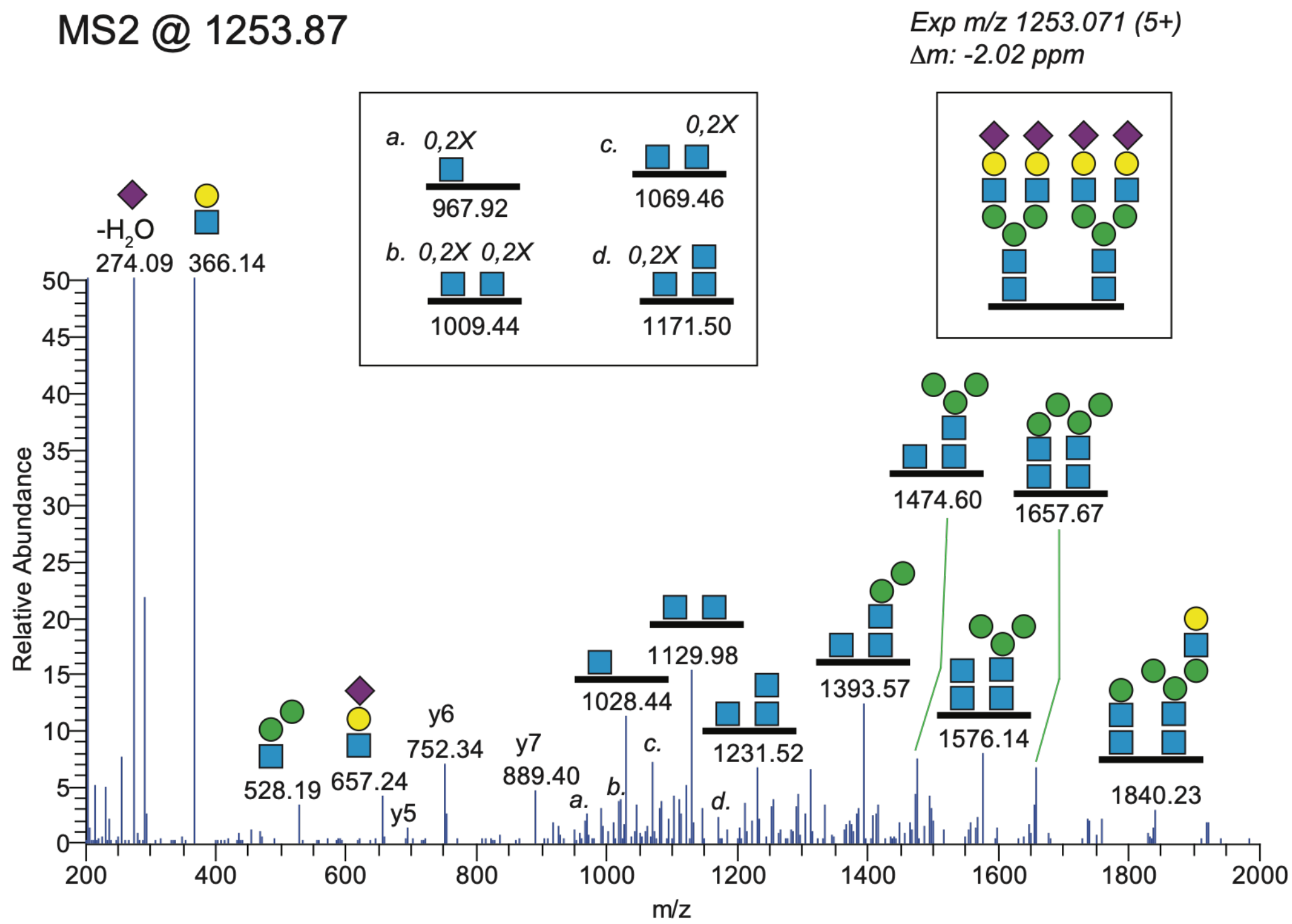

Supporting Fig. 8. Doubly N-glycosylated peptide [amino acids 240-256] from hemopexin in a control sample. The annotated MS2 spectrum demonstrates that two di-sialylated biantennary N-glycans are attached to Asn-240 and Asn-246. The peptide sequence is NGTGHGNSTHHGPEYMR (NXS/T motifs are underlined). All annotated Y-ions are doubly charged. The highest intensity ion peaks are annotated, which often is not the monoisotopic one. See insert for the *a-d* annotation of ^{0,2}X ring-cleavage ions of the N-glycosidically linked HexNAc of Y1. The experimental *m/z* of the precursor ion had a mass accuracy of -2.02 ppm.

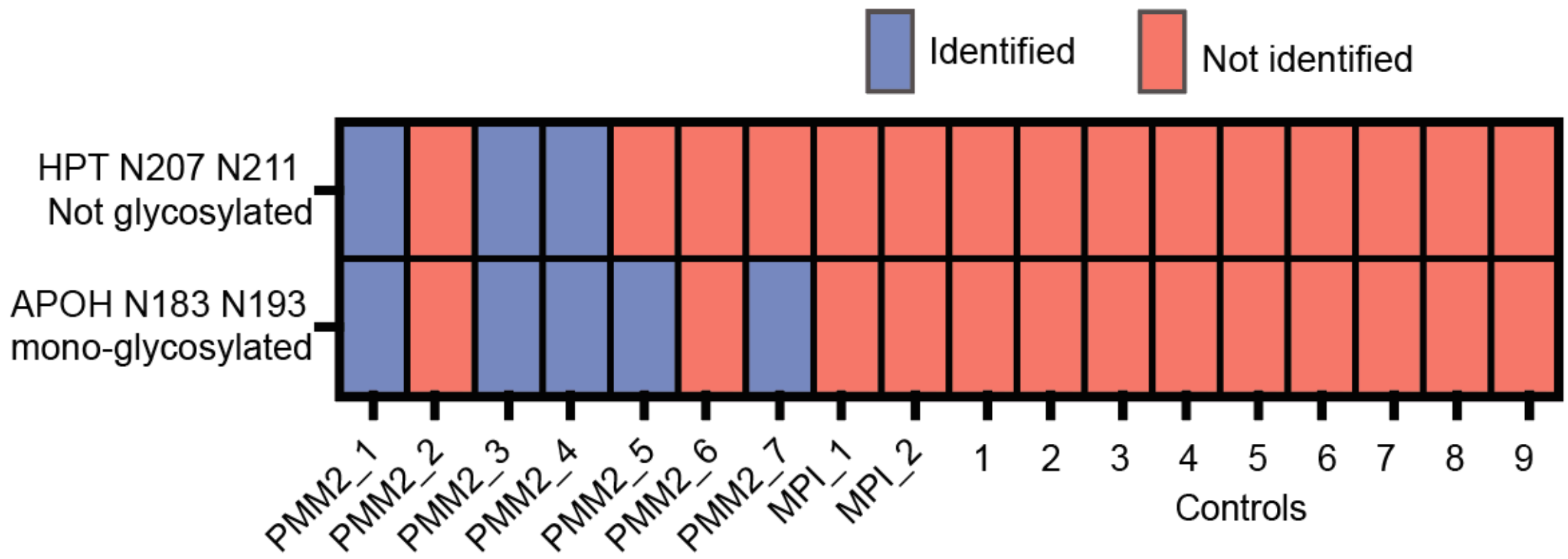

Supporting Fig. 9. Identification of a non-glycosylated peptide containing the dual Asn-207 and Asn-211 glycosylation sites from haptoglobin (HPT), and identification of a mono-glycosylated glycopeptide containing the Asn-183 and Asn-193 glycosylation sites from apolipoprotein H (APOH).
