# Supplementary material for: Integrated glycoproteomics reveals site-specific N-glycosylation defects in phosphomannomutase two congenital disorder of glycosylation
Source: J Biol Chem. 2026 Aug 19;302(10):113469. doi: 10.1016/j.jbc.2026.113469 (PMC13597197; doi:10.1016/j.jbc.2026.113469)
Supplement: Supplementary file 2 [file mmc2.docx]

**Supplementary Table 1 Control individuals**

| **Control ID** | **Sex** | **Sample matrix** | **Age at sampling** |
| --- | --- | --- | --- |
| 1 | F | plasma | 4 mth |
| 2 | F | plasma | 9 mth |
| 3 | F | plasma | 2 y |
| 4 | F | plasma | 3 mth |
| 5 | F | plasma | 9 mth |
| 6 | M | plasma | 4 y 10 mth |
| 7 | M | plasma | 5 y 9mth |
| 8 | M | plasma | 13 y |
| 9 | M | plasma | 58 y |
